# Lipidomic profile of meningiomas harboring different NF2 mutation status

**DOI:** 10.1007/s11306-026-02399-4

**Published:** 2026-05-07

**Authors:** Joanna Bogusiewicz, Ivana Stanimirova, Magdalena Gaca-Tabaszewska, Paulina Szeliska, Krystyna Soszyńska, Anna Majdańska, Agata Ryfa, Alicja Bartoszewska-Kubiak, Jacek Furtak, Marcin Birski, Marek Harat, Barbara Bojko

**Affiliations:** 1 Department of Pharmacodynamics and Molecular Pharmacology, Faculty of Pharmacy, Collegium Medicum in Bydgoszcz, Nicolaus Copernicus University in Torun, Jurasza 2, 85-089 Bydgoszcz, Poland; 2 Institute of Chemistry, University of Silesia in Katowice, Szkolna 9, 40-006 Katowice, Poland; 3 Clinical Genetics and Molecular Pathology Laboratory, Department of Medical Analytics, 10th Military Research Hospital and Polyclinic, Powstańców Warszawy 5, 85-681 Bydgoszcz, Poland; 4 Department of Basic Research, Faculty of Medicine, Bydgoszcz University of Science and Technology, Al. prof. S. Kaliskiego 7, 85-796 Bydgoszcz, Poland; 5 Medical Faculty, Bydgoszcz University of Science and Technology, Al. prof. S. Kaliskiego 7, 85-796 Bydgoszcz, Poland; 6 Department of Neurosurgery, 10th Military Research Hospital and Polyclinic, Powstańców Warszawy 5, 85-681 Bydgoszcz, Poland

**Keywords:** Meningioma, Brain tumor, SPME, Lipidomics, NF2, Merlin

## Abstract

**Introduction:**

Meningiomas are mainly benign brain tumors, but they can evolve to higher grades. The phenomena of these changes are not well-known. Therefore, more basic research is needed. This study attempted to assess the lipidome profile in meningiomas harboring different NF2 mutation statuses (wildtype and mutated). Solid-phase microextraction (SPME) probes were used to sample and extract the metabolites and reduce the invasiveness of lipidomic analysis.

**Objectives:**

This study aimed to select the set of lipids distinguishing meningiomas with different genotypes using two chromatography methods (hydrophilic interaction chromatography (HILIC) and reversed-phase chromatography (RPLC) in two ionization modes.

**Methods:**

Brain tumors were obtained during neurosurgical procedures. Then, sampling using SPME fibers was performed directly after the lesion excision. After collecting the whole batch of samples, desorption using an isopropanol-methanol solution was performed. Subsequently, instrumental analysis was carried out using liquid chromatography coupled with high-resolution mass spectrometry. The remaining part of the lesion was stored as paraffin tissue blocks, and then genetic testing was performed to determine the presence of mutations in the NF2 gene.

**Results:**

Genetic profiling of meningiomas revealed that most lesions had a mutation in the NF2 gene. A wide range of analytes was extracted from the studied tumors using SPME probes. A set of 34 lipids was selected as crucial metabolites in tumor differentiation. A combination of analytes detected in more than one analysis mode demonstrated higher sensitivity and specificity compared to the individual models and increased the differentiation of mutant and wildtype samples.

**Conclusions:**

SPME coupled liquid chromatography and mass spectrometry, can be successfully applied to the screening of lipids in meningiomas with different NF mutation statuses.

**Graphical abstract:**

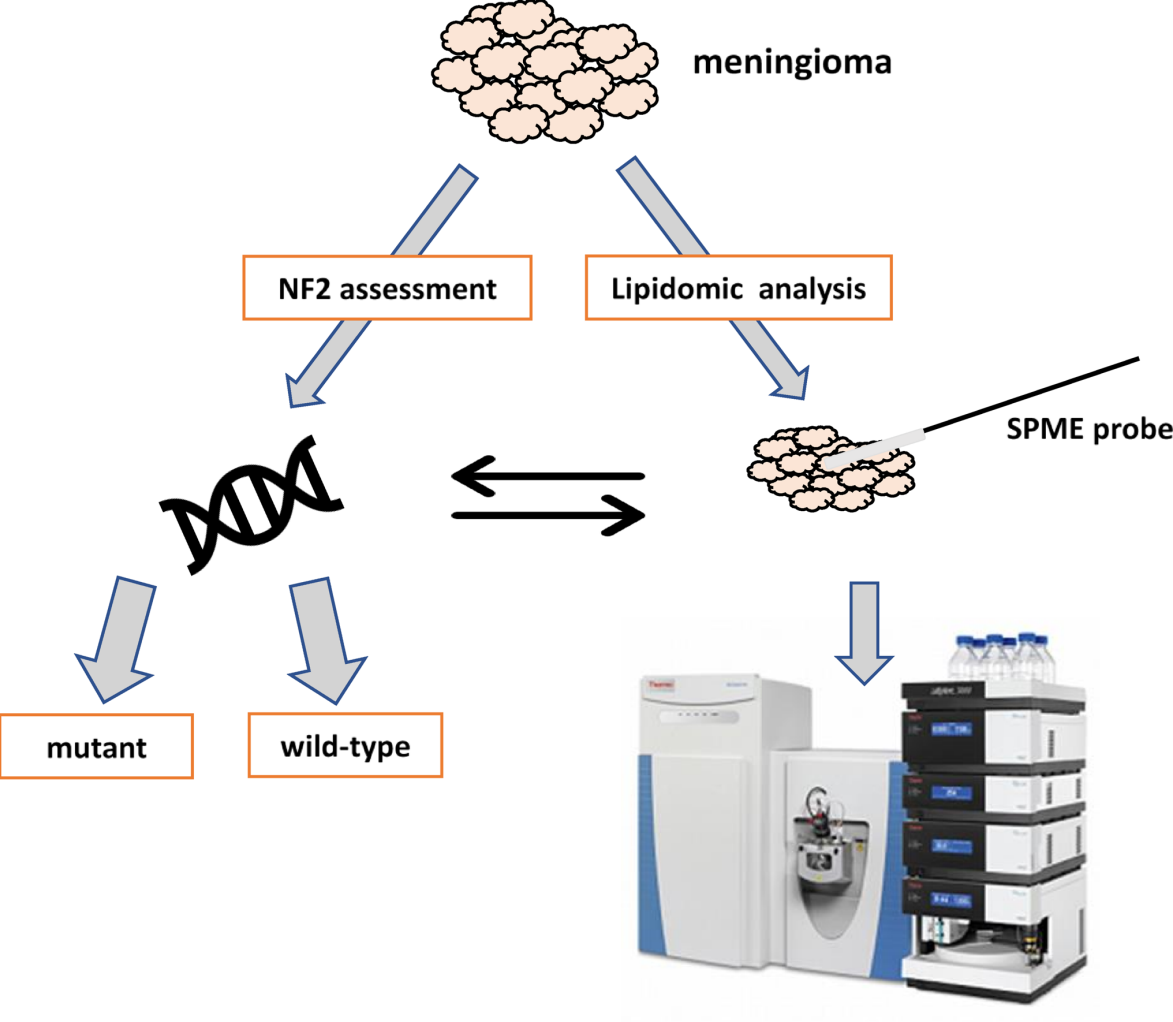

**Supplementary Information:**

The online version contains supplementary material available at 10.1007/s11306-026-02399-4.

## Introduction

Primary brain tumors are classified by World Health Organization (WHO) recommendations (Louis et al., 2021). Most of them are benign lesions, but some can evolve into II or III-grade tumors (Nowosielski et al., 2017). Surgical removal is the best treatment option, although, in some cases, it cannot be entirely removed or is malignant, so chemotherapy or radiotherapy must be applied (Gupta et al., 2018; Nowosielski et al., 2017). Therefore, basic research in the direction enabling understanding of the relationship between genetic mutations, their translation to molecular biology, and, subsequently, the impact on mechanisms behind the sudden increase of malignancy is of great importance (Nowosielski et al., 2017).

The WHO recommendation implies the high importance of genetic tests in diagnosing brain tumors (Louis et al., 2021). The most common genetic aberration in meningioma patients is a mutation in the NF2 (Lee et al., 2019). The protein encoded by NF2 is merlin, a member of a moesin-ezrin-radixin-like protein family, and it regulates cell adhesion, proliferation, and survival signaling and suppresses tumorigenesis (Lee et al., 2019). There are also mutations observed less often, such as mutations in Akt murine thymoma viral oncogene homolog 1 (ATK1), Kruppel-like factor 4 (KLF4), tumor necrosis factor receptor-associated factor 7 (TRAF7) in NF2 wildtype samples (Nowosielski et al., 2017).

Molecular markers were proposed in diagnosis, but are still not correlated with tumor type, progression, and clinical outcomes. Therefore, further research in this direction is conducted, and genetics testing is enriched with metabolite profiling (Gaca-Tabaszewska et al., 2022). An important group of metabolites are lipids due to their building and signaling role (Pan et al., 2021). One of the most often used analytical platforms in lipidomic profiling is liquid chromatography coupled with mass spectrometry (LC-MS) (Cajka & Fiehn, 2016). Liquid chromatography coupled with mass spectrometry (LC-MS) is mainly used for analyte separation but different approaches can be applied (Cajka & Fiehn, 2014). Reversed-phase liquid chromatography (RPLC) enables the separation of lipids based on the length of acyl chains and saturation status. Hydrophilic interaction chromatography (HILIC) and normal phase chromatography (NPLC), on the other hand, separate lipids based on lipid group affiliation (e.g., glycerolipids, glycerophospholipids, sphingolipids) (Cajka & Fiehn, 2014). Nevertheless, apart from instrumental analysis, sample preparation also has to be conducted. Usually, collected tissue has to be homogenized, and then the analytes are extracted. The most popular extraction method for lipid analysis is liquid-liquid extraction using chloroform (Folch method) or methyl tert-butyl ether (MTBE) (Cajka & Fiehn, 2014). These procedures are time and labor-consuming, but provide wide-range coverage of lipids. An alternative fast and easy approach is solid phase microextraction (SPME), which combines sampling and extraction in one step. Consequently, SPME offers the features unavailable for traditional methods, including extraction from intact tissue, spatial analysis, and in vivo studies, while compromising the range of extracted metabolites. This sample preparation method is referred to as a chemical biopsy due to the sampling of analytes without tissue consumption (Bogusiewicz et al., 2021). In brain studies, SPME was applied for the analysis of neurotransmitters in rats and monkeys, and for the pilot study of metabolomic and lipidomic profiling of the human brain in vivo (Bogusiewicz et al., 2021; Cudjoe et al., 2013; Lendor et al., 2019). Apart from healthy tissue, SPME was also used for brain tumor analysis (Bogusiewicz et al., 2022, 2025; Goryńska et al., 2022).

Combining SPME probe characteristics and the need for developments in meningioma diagnosis, this study aimed to assess the lipidome profile obtained using chemical biopsy in tumors with different NF2 mutation statuses. Tests using different ionization and chromatographic separation modes were used to explore the possibility of selecting the minimum number of lipids that could be successfully used to predict the genotype of meningioma.

## Materials and methods

### Chemicals and materials

Isopropanol, methanol, water, acetonitrile, ammonium acetate and acetic acid used in this research were liquid chromatography-mass spectrometry (LC-MS) grade and were purchased from Merck (Warsaw, Poland). External calibrant Pierce LTQ Velos ESI Positive Ion Calibration Solution was obtained from Thermo Scientific (San Jose, CA, USA), and fibers coated with an octadecyl (C18) were kindly provided by Supelco (Bellefonte, PA, USA). SPLASH™ LIPIDOMIX™ Mass Spec Standard was purchased from Merck (Warsaw, Poland).

### Biological material

Brain tumors were obtained during neurosurgical procedures in the 10th Military Research Hospital and Polyclinic in Bydgoszcz. A detailed characterization of the patients included in these experiments is provided in the supplementary materials (Table S1). The study was approved by the Bioethical Committee in Bydgoszcz (KB 628/2015).

### Histological data and genetic test results

Tumor specimens were formalin-fixed and paraffin-embedded. All samples were classified by histopathological examination and graded according to WHO 2016 guidelines. The NF2 mutation was detected using the multiplex ligation-dependent probe amplification (MLPA) method following the manufacturer’s protocol. More details were given in the Supplementary Materials.

### Chemical biopsy (Solid-phase microextraction) protocol

SPME probes with 7 mm C18 sorbent were used to sample the excised brain tumors. The exact protocol was described elsewhere (Bogusiewicz et al., 2022). Briefly, the sorbent was preconditioned in a methanol-water solution, 1:1,v/v. Then, the probe was inserted into the brain tumor for 30 min. Subsequently, the probe was removed from the sample, washed, and stored at −30 °C until instrumental analysis. Finally, the analytes were desorbed in 150 µl of isopropanol: methanol (1:1 v/v) using silanized inserts during 1 h desorption under agitation at 850 rpm. Extraction blanks were also prepared (probes underwent all of SPME protocol steps, omitting extraction).

### Liquid chromatography-high resolution mass spectrometry (LC-HRMS) analysis

LC-HRMS (Q Exactive Focus, Thermo Scientific, Bremen, Germany) was used for instrumental analysis.

The HILIC (H) parameters were as follows: eluent A—5 mM ammonium acetate in water; eluent B—acetonitrile; gradient—0–2 min at 96% B, gradual decrease of B until 80% B at 15.0 min, and 15.1–21.0 min at 96% B; SeQuantZIC-cHILIC (Merck, Poznań, Poland) 3 μm 100 × 2.1 mm column; mobile phase flow rate—0.4 mL/min; oven temperature—40 °C; and injection volume—10 µL. HILIC-HRMS analysis was conducted in positive (Hp) and negative (Hn) ion modes. The MS parameters were given elsewhere (Bogusiewicz et al., 2020).

The RPLC (R) mobile phase consisted of: eluent A: methanol: water, 40:60 with 10 mM ammonium acetate and 1 mM acetic acid, and eluent B: isopropanol: methanol, 90:10 with 10 mM ammonium acetate and 1 mM acetic acid. Mobile phase was pumped with the flow rate: 0.2 mL/min, and the gradient was as follows: 0 min – 20% B; 1.0 min – 20% B; 1.5 min – 50% B; 7.5 min – 70% B; 13.0 min – 95% B; 17.0 min – 95% B; 17.1–23.0 min – 20% B. XSelect C18 Column (Waters, Warsaw, Poland), 3.5 μm, 2.1 mm x 75 mm. The oven temperature was set at 55 °C and the injection volume at 10 µL. RPLC-HRMS analysis was conducted in positive (Rp) and negative (Rn) ion modes. The MS parameters were given elsewhere (Bogusiewicz et al., 2020).

Samples were run using LC-HRMS in full scan mode with fragmentation of ions from an inclusion list. The inclusion list was prepared based on the preliminary LC-HRMS analysis of the pooled quality control sample run in full scan with discovery fragmentation. Identification was based on LipidSearch 4.1.30 (Thermo Fisher Scientific, San Jose, CA, USA) library search, with mass accuracy of <3ppm. The MS parameters were given elsewhere (Bogusiewicz et al., 2020). Identification was accepted when the identification grade was A (identification of the whole lipid class and fatty acids) or B (identification of at least a lipid group). M-score was set at 5.0; the confirmation of lipid structure was accepted when it was present in all QC samples or at least half of the study group samples.

The MS was externally calibrated every 72 h, and mass accuracy was below 2ppm. Tumor samples in the sequence were randomized, and pooled quality controls (QC) were analyzed every 10–12 injections. Apart from pooled QC, which consisted of extracts obtained from different types of tumors, extraction blanks, treated as a negative control, were run. Extraction blanks were obtained by the desorption of the SPME probe treated in the same way as the one that was inserted into the sample, but with the omission of the extraction step. Online monitoring of samples was possible because the desorption solvent was spiked with SPLASH™ LIPIDOMIX™ Mass Spec Standard solution. This solution was added to the desorption solvent so it was possible to use it only for analysis monitoring.

### Data preprocessing and supervised multivariate analysis

Data acquisition was performed using dedicated Thermo Scientific software: Xcalibur 4.2 (Thermo Fisher Scientific, San Jose, CA, USA). The data for the lipidomic studies were processed using LipidSearch 4.1.30 (Thermo Fisher Scientific, San Jose, CA, USA) with its accuracy set to 3 ppm and intensity threshold set to 10,000. The searched ion adducts included H^+^, NH_4_^+^, Na^+^, H^–^, and CH3COO^–^ The obtained results were then filtered using the following parameters: for extraction quality control (QC), an area coefficient of variation (CV) below 30% and not equal to 0; the QC: extraction blank area ratio above 2.0; and a peak quality factor above 0.85 for at least one of the studied groups. Further information on these search parameters has been detailed elsewhere (Bogusiewicz et al., 2022). The Principal Component Analysis (PCA) presenting QC was given in the Supplementary Materials (Fig. S1). After filtering the results, the peak areas for all detected lipids were normalized by the summary peak area of the probe, followed by autoscaling to the unitary standard deviation for each data variable (lipid). Then, a supervised multivariate analysis including the discriminant version of partial least squares (PLS-DA) regression combined with a variable selection procedure was performed for the discrimination of wild-type meningioma samples (NF2wt) from those with the NF2 mutation (NF2mt).

The PLS-DA was combined with a bootstrapping procedure to estimate the quality of the models with all selected variables. The representativeness of the model set, while avoiding the possibility of including outliers, was guaranteed using the Kennard and Stone algorithm applied for each of the two groups separately with all variables (lipids). The model set was balanced, including 23 samples of each group, estimated as 75% of the less numerous group (31 NF2wt samples). The test set included the remaining samples (8 NF2wt and 27 NF2mt samples). The lipids important for group differentiation were chosen based on the selectivity ratio (SR) value obtained as the ratio of explained variance to the residual variance for a variable (lipid) after a target projection transformation (Kvalheim, 2010). For each (out of 1,000) bootstrap sample generated from the original model set by re-sampling with re-placement, a PLS-DA model of a definite complexity selected by leave-one-sample-out cross-validation was performed, and SRs for variables were calculated. The variables with average SR values over a given cut-off value were kept as important in the final model. The cut-off value of SR for each model was determined using the discriminating variable test (DIVA) and the SR plot (Rajalahti et al., 2009). DIVA is a nonparametric test that allows for the relation of the mean correct classification rate (MCCR) for variables in a given SR interval and for determining discriminatory ability in the entire SR range. The average value of the area under the receiver operating curve (AUC) was used as a figure of merit describing the model’s performance. At the same time, AUC, sensitivity, and specificity for the test set were calculated to describe the model’s predictive ability. A detailed scheme of the data analysis was presented elsewhere (Ząbek et al., 2015).

All calculations were performed with MATLAB 2017 on a personal computer (Intel(R), Core(TM) i7-8550U CPU @ 1.80 GHz, 2.00 GHz with 32GB RAM) using the Microsoft Windows 10 operating system.

## Results

Analysis of meningiomas regarding the mutation status of the NF2 gene revealed that 71% of studied brain tumors were wild-type, while 29% of them did not have this change. Subsequently, lipidomic profiling was performed using two chromatography types, RPLC and HILIC, in both ionization modes.

Application of Hp enabled the detection of 74 lipids, which were classified as four SBP, seven SM, two PS, 22 PE, 12 PC, five LPC, 10 HexCer, one Cer, and 11 acylcarnitines (Table S2). The highest number of different species of PC and PE was detected, and these phospholipids were also the most abundant analytes based on their summary peak areas. It was observed that phospholipids such as LPC, PC, PS, and PE were mainly lower in NF2mt samples, while plasmogens of PC and PE were higher (Table S2). In the SM group, selecting one trend of change was impossible (Table S2). Interestingly, it was observed that HexCer species were slightly higher in NF2mt tumors, but only one analyte was significantly changed (Table S2). Acylcarnitines were not significantly changed, but the trend of levels in NF2mt tumors was observed (Table S2).

**Fig. 1:**
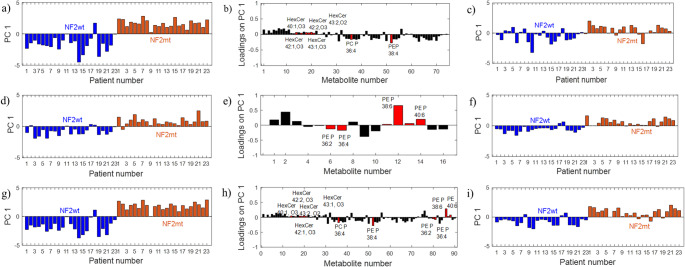
PLS-DA with TP for metabolites obtained from the Hp and Hn: **a**) TP scores vector from the model with all Hp metabolites, **b**) TP loadings vector with the selected Hp metabolites (Table 1), **c**) TP scores vector from the model with Hp selected metabolites by SR, **d**) TP scores vector from the model with all Hn metabolites, **e**) TP loadings vector with the selected Hn metabolites (Table 1), **f**) TP scores vector from the model with Hn selected metabolites by SR, **g**) TP scores vector from the model with all Hp + Hn metabolites, **h**) TP loadings vector with the selected Hp + Hn metabolites (Table 1), **i**) TP scores vector from the model with Hp + Hn selected metabolites by SR. *Hn* HILIC in negative mode, *Hp* HILIC in positive mode, *SR* selectivity ratio, *TP* target projection

Next, chemometric analysis was performed. The values in Table 1 show that the model built for all lipids obtained using the Hp presents good predictive ability (AUC_test_=0.99). This model’s sensitivity of 87.5% indicates a very good prediction; three NF2mt samples were incorrectly predicted as NF2wt, resulting in a specificity of 88.9%. Additionally, the PLS-DA model (AUC_test_=0.78) using only eight lipids selected based on SR showed a very low specificity of 44.4%, which means that fifteen NF2mt samples were wrongly recognized as wild-type samples. The model has a relatively good sensitivity of 75.0% (Fig. 1, Table S6).

**Table 1:** The average AUC values (± uncertainty in the AUC estimation) for the model set (AUC_model_) and the AUC values for the test set obtained from PLS-DA with all HILIC variables and variables selected using the SR approach

Model	PLS-DA (complexity	AUC_model_	AUC_test_	Sensitivity [%]	Specificity [%]	Cut-off value of SR (MCCR [%])	Variables
Hp	4	0.99 ± 0.01	0.89	87.5	77.8	− (88.6)	all (74)
Hn	6	0.98 ± 0.01	0.89	87.5	88.9	− (80.0)	all (16)
Hp	4	0.84 ± 0.06	0.78	75.0	44.4	0.40 (51.4)	(8) HexCer 40:1,O3; HexCer 42:1,O3; HexCer 42:2, O3; HexCer 43:1, O3; HexCer 43:2, O2; HexCer 44:2, O2; PC P-36:4; PE P-38:4
Hn	6	0.96 ± 0.02	0.89	87.5	88.9	0.20 (80.0)	(6) PE P 36:2; PE P 36:4; PE 38:6; PE P 38:6; PE 40:6; PE P-40:6
Hp + Hn	4	0.99 ± 0.01	0.92	87.5	88.9	− (88.6)	all (90)
Hp + Hn	4	0.96 ± 0.02	0.87	87.5	74.1	0.20 (77.1)	(14)

Sensitivity and specificity for the test set are also presented. *Hp* HILIC in positive mode, *Hn* HILIC in negative mode

Changing the ionization mode to negative allowed the detection of 16 lipid ions classified as 14 PE, one PS, and one PI. Similarly to previous observations, phospholipids were lower, but some plasmalogens were detected at higher levels in NF2mt tumors (Table S3). Compared to the positive ionization mode, the model (AUC_test_=0.89) for the Hn lipids showed the same sensitivity of 87.5%, but a higher specificity of 88.9%. The model with the selected six phospholipids had the same sensitivity and specificity as the model with all lipids (Fig. 1; Table 1).

A model using all lipids (90) obtained using Hp and Hn presented similar prediction capability, but not better, than the predictions for individual models (Table 1). Furthermore, the combined model based only on 14 lipids selected based on SR showed a little worse specificity than the individual Hn model with six lipids.

Rp enabled the detection of 95 lipid ions, corresponding to 56 lipid species classified as one Cer, 8 HexCer, two CheE, five PC, 14 PE, one PI, three PS, one SM, two SBP, one acylcarntine, five DG, one MG and 12 TG. The trend was observed that phospholipids were lower in NF2mt samples, while plasmalogens of PC and PE were higher (Table S4). HexCer was at a lower level in NF2mt meningiomas, but only HexCer 44:2,O2 was significantly altered (*p* < 0.05). Sphingomyelins were altered, but there was difficulty in finding the trend of change. In the group of glycerides, no significant alterations were observed; there was only a weak trend of higher TG levels in wildtype samples (Table S4).

**Fig. 2:**
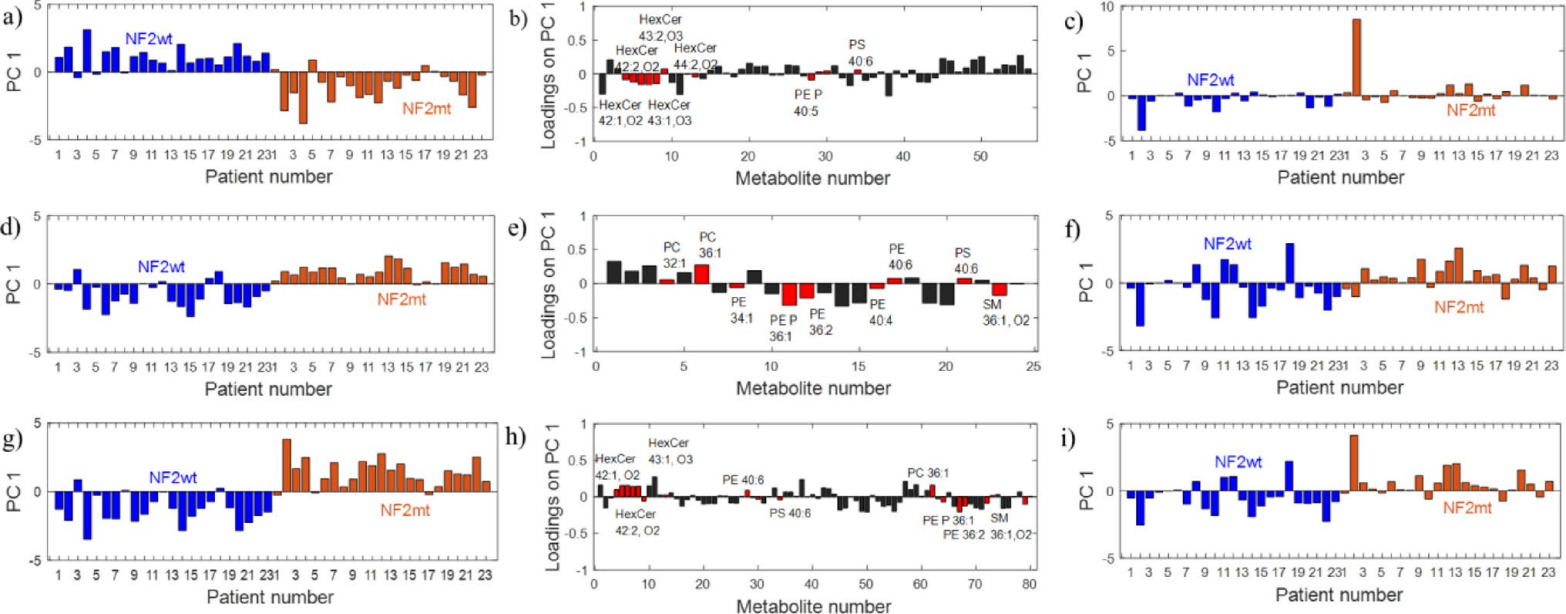
PLS-DA with TP for metabolites obtained from the Rp and Rn: **a**) TP scores vector from the model with all Rp metabolites, **b**) TP loadings vector with the selected Rp metabolites (Table 2), **c**) TP scores vector from the model with Rp selected metabolites by SR), **d**) TP scores vector from the model with all Rn metabolites, **e**) TP loadings vector with the selected Rn metabolites (Table 2), **f**) TP scores vector from the model with Rn selected metabolites by SR, **g**) TP scores vector from the model with all Rp + Rn metabolites, **h**) TP loadings vector with the selected Rp + Rn metabolites (Table 2), **i**) TP scores vector from the model with Rp + Rn selected metabolites by SR. *Rp* RPLC in positive mode, *Rn* RPLC in negative mode, *SR* selectivity ratio, *TP* target projection

The PLS-DA model (AUC_test_=0.79) using all lipids was characterized by a high sensitivity of 87.5%, but a low specificity of 44.4% (Table 2). Eleven lipid ions were chosen based on the SR approach and were important to distinguish between NF2wt and NF2mt samples (Table 2; Fig. 2). Compared to the model with all 56 lipids, the model with the selected 11 lipids (AUC_test_=0.54) presented a slightly lower sensitivity of 75.0% and a specificity of 40.1% (Table 2).

**Table 2:** The average AUC values (± uncertainty in the AUC estimation) for the model set (AUC_model_) and the AUC values for the test set obtained from PLS-DA with all RPLC variables and variables selected using SR approach

Model	PLS-DA (complexity	AUC_model_	AUC_test_	Sensitivity [%]	Specificity [%]	Cut-off value of SR (MCCR [%])	variables
Rp	3	0.98 ± 0.02	0.79	87.5	44.4	− (54.3)	all (56)
Rn	3	0.97 ± 0.02	0.86	87.5	59.2	− (65.7)	all (24)
Rp	1	0.68 ± 0.09	0.54	75.0	40.1	0.20 (48.6)	(11) HexCer 42:1;O2, HexCer 42:2;O2, HexCer 43:1;O3, HexCer 43:2;O3 HexCer44:2;O2, PC 34:0, PE P-40:5, PE 40:6, PE P 40:6, PS 40:6
Rn	1	0.79 ± 0.06	0.71	75.0	40.1	0.10 (48.6)	(9) PC 32:1, PC 36:1, PE P-36:1, PE 34:1, PE 36:2, PE 40:4, PE 40:6, PS 40:6, SM 36:1;O2
Rp + Rn	3	0.99 ± 0.01	0.85	87.5	51.8	− (60.0)	all (80)
Rp + Rn	2	0.86 ± 0.05	0.70	75.0	33.3	0.20 (42.8)	(20)

Sensitivity and specificity for the test set are also presented. *Rp* RPLC in positive mode, *Rn* RPLC in negative mode

Changing the ionization mode to negative allowed the detection of 24 lipid. These analytes were classified as three CER, four PC, 11 PE, two PS, one PI, and three SM. Similar to the observation of analytes using other modes, phospholipids with the expectation of plasmalogens were downregulated in NF2mt (Table S5). The trend of changes for SM was not obvious (Table S5).

The discriminant model using all these analytes (AUC_test_=0.86) allowed for differentiated tumors with different NF mutation statuses with a high sensitivity of 87.5%, but with a relatively low specificity of 59.2% (Table 2). The model built for the selected lipids was characterized by a sensitivity of 75.0% and a specificity of 40.1% (Fig. 2; Table 2).

A combination of the lipids (80) obtained in positive and negative modes gave a model with similar predictive abilities (AUC_test_ = 0.85) as the model for the lipids determined by Rp and Rn with only slightly better predictions compared to the model using the lipids in the positive mode (Table 2). Looking at the predictive capabilities of models with fewer variables, the model (AUC_test_=0.70) with 20 lipids determined in Rp and Rn showed a sensitivity of 75.0% and a very poor specificity of 33.3% (Table 2).

**Table 3:** The average AUC values (± uncertainty in the AUC estimation) for the model set (AUC_model_) and the AUC values for the test set obtained from PLS-DA of various combinations HILIC and RPLC variables determined in negative and positive modes and variables reduced using the SR approach

Model	PLS-DA (complexity)	AUC_model_	AUC_test_	Sensitivity [%]	Specificity [%]	Cut-off value of SR (MCCR [%])	Variables (number of variables)	
Hp + Rp	4	0.99 ± 0.01	0.96	100.0	85.2	− (88.6)	all (130)	
Hp + Rp	2	0.82 ± 0.06	0.69	75.0	40.1	0.20 (48.6)	(19)	
Hp + Rn^+^	4	0.99 ± 0.01	0.89	87.5	77.8	− (80.0)	all (98)	
Hp + Rn	5	0.98 ± 0.02	0.87	75.0	77.8	0.20 (60)	(23)	
Hn^#^+Rn	2	0.98 ± 0.02	0.92	87.5	81.5	− (82.8)	all (40)	
Hn + Rn	3	0.96 ± 0.03	0.94	87.5	88.9	0.10 (88.6)	(15)	
Hn + Rp	7	0.99 ± 0.01	0.92	87.5	70.4	− (74.3)	all (72)	
Hn + Rp	5	0.99 ± 0.01	0.92	75.0	92.6	0.20 (88.6)	(17)	
Hp + Hn+Rp	2	0.99 ± 0.01	0.95	100.0	74.1	− (80.0)	all (146)	
Hp + Hn+Rp	4	0.98 ± 0.02	0.94	87.5	85.2	0.20 (85.7)	(25)	
Hp + Hn+Rn^+^	4	0.99 ± 0.01	0.92	87.5	88.9	− (88.6)	all (114)	
Hp + Hn+Rn	4	0.98 ± 0.02	0.94	87.5	88.9	0.20 (88.6)	(23)	
Hn + Rp+Rn	5	0.99 ± 0.01	0.92	87.5	74.1	− (77.1)	all (96)	
Hn + Rp+Rn	3	0.98 ± 0.02	0.96	87.5	81.5	0.10 (82.8)	(26)	
Hp + Hn+Rp + Rn	2	0.99 ± 0.01	0.95	100.0	74.1	− (80.0)	all (170)	
Hp + Hn+Rp + Rn	4	0.99 ± 0.01	0.95	87.5	85.2	0.10 (85.7)	(34)	

Sensitivity and specificity for the test set are also presented. *Hp* HILIC in positive mode, *Hn* HILIC in negative mode, *Rp* RPLC in positive mode, *Rn* RPLC in negative mode

From a practical point of view, using either the HILIC or RPLC for analysis is preferred. Comparing the results from both methods, the discriminant model differentiating the NF2wt meningiomas from those of NF2mt was the best in terms of predictive capabilities when using only six lipids determined by the Hn. An equally good model in terms of predictive abilities is the model with all 16 lipids determined by Hn (Table 1). The model using 20 lipids selected based on SR, analyzed using Rp + Rn, presented a much worse predictive ability (Table 2).

In the next step, combinations of lipids determined by two different chromatography methods and ionization modes in meningioma differentiation were tested. The merit data are presented in Table 3. The best-combined model (AUC_test_=0.99) with the highest sensitivity of 100.0% and a specificity of 85.2% was obtained for all 130 lipids determined by Hp + Rp (Table 3). There are several combined models with a sensitivity of 87.5% and a specificity of 88.9%, indicating that in these cases, one NF2wt meningioma sample was incorrectly recognized as an NF2mt, and three NF2mt samples were incorrectly recognized as NF2wt (Table 3). The same samples were incorrectly recognized in these models, suggesting that another factor, which was not analyzed within this study, may influence lipidome composition.

The minimal set of lipids that gave the best discrimination between NF2wt and NF2mt samples, regardless of the chromatographic method and ionization mode, were 15 lipids (Table 3), which were determined by a combination of Hn + Rn (Table 3). This set of lipids allowed to discriminate the groups of meningioma samples with a specificity of 88.9% and sensitivity of 87.5%.

## Discussion

A mutation in the NF2 gene was observed in the majority of studied meningiomas, which correlates with literature reporting that this aberration can be detected in over 60% of patients (Lee et al., 2019). A wide range of lipids, including long-chain acylcarnitines, phospholipids, sphingolipids, and glycerides, were detected in the experiment presented herein. Previous studies on brain tumors showed a similar range of analytes (Bogusiewicz et al., 2022; Jarmusch et al., 2016; Yu et al., 2020). It should be noted that detected analytes formed common adducts such as [M + H]^+^, [M–H]^−^, or [M+NH4]^+^, but also less common [M + Na]^+^ were often observed (Tables S7–S14). These adducts are less common but can be observed in biological samples due to the high abundance of sodium in biological samples (Kruve et al., 2013). Moreover, apart from the even-chain fatty acids common in animals, odd-chain fatty acids were observed. These lipids are typically present in human samples only at low abundance (Jenkins et al., 2015). However, their occurrence in the present study may be attributed to several factors: (i) tumor type-specific occurrence, (ii) enhanced extraction efficiency of unbound or weakly protein-bound analytes performed by solid-phase microextraction (SPME), or (iii) ion-source fragmentation or other analytical artifacts. Thus, further investigation is required to distinguish between these possibilities.

Lipidomic analysis showed that most of the phospholipids were decreased in NF2-mutant meningiomas, which is surprising given merlin’s role as a suppressor in the hippo pathway regulating the proliferation and growth of cells (Xu et al., 2024). Thus, the lack of merlin is expected to be related to cancer growth, which, at the same time, is associated with increased phospholipid levels reflecting lipid membrane remodelling, higher energy demand, and increased lipid uptake (Bogusiewicz et al., 2022; Cheng et al., 2016; Delmas et al., 2025; Martin-Perez et al., 2022; Melone et al., 2018; Sioris et al., 2024). It can be hypothesized that the increase in phospholipid level was not observed due to the benign character of the studied tumors; however, this needs to be investigated in further studies. It should also be noted that many PC and PE plasmalogens were extracted, which is in line with previous reports that they can constitute 20–50% of brain phospholipid mass (Ferreri et al., 2023) (Table S2, S3, S4, S5). These lipids are highly sensitive to acids and redox reactions, making them unstable during sample preparation. SPME may help preserve these analytes by quenching enzymatic reactions upon sorbent binding, similarly to its effect on unstable oxylipins, allowing for their extraction (Bogusiewicz et al., 2021; Napylov et al., 2020). Notably, certain plasmalogens (e.g., PE P-38:6, PE P-40:6, PC P-34:1, PC P-36:4) were elevated in NF2-mutant samples and strongly influenced tumor differentiation (Tables 1 and 2, S6). Their antioxidant activity may play a role in cancer development, making these analytes potential indicators of early changes (Messias et al., 2018). Lysophospholipids, which, among others, take part in signalling processes and cell membrane fluidity through the Lands cycle, were not significantly altered (Hishikawa et al. 2008). However, chemometric models identified them as discriminatory lipids (Tables 1 and 2). The alterations in this group of lipids were reported before. For instance, higher LPC levels were observed in high-grade gliomas and IDH1/2 wildtype lesions, which have poorer outcomes than low-grade and IDH1/2 mutant tumors (Bogusiewicz et al., 2022). It could also indicate that cancerous changes and the lack of suppression in the hippo pathway disrupt lipid turnover in cell membranes. Notably, PS 40:6 was identified by chemometric models as a discriminatory lipid explaining potential changes in cell signalling, membrane dynamics, and apoptosis (Table 2) (Furuta & Zhou, 2023; Kaynak et al., 2022). PS in cells is responsible for apoptosis, so the observed trend of its slightly lower levels (*p* > 0.05) in NF2mt can be related to a lower ability for apoptosis (Table S2–S5).

Sphingolipid metabolism integrates three pathways, including sphingomyelin synthesis, salvage, where sphingosine is produced, and modified ceramide hydrolysis (hexosylceramide formation), all known to influence cancer cell growth, migration, autophagy, and apoptosis, (Li et al., 2022). In this study, ceramide derivatives such as sphinganine and hexosylceramide were selected as discriminatory, showing their impact on tumor metabolism (Tables 1 and 2). For instance, they participate in apoptosis, which can be induced by the cleavage of procaspase-3, (Farley et al., 2024). Although no uniform trend of change was observed for sphingomyelins, these lipids contribute to lipid raft formation and serve as precursors for bioactive ceramides, potentially affecting apoptosis and other key cellular processes (Hirano et al., 2022). Moreover, these analytes are the major source of ceramides, one of the most bioactive lipids (Goñi, 2022).

Among extracted glycerides, triglycerides are the biggest group. It should also be noted that TG serve as reservoirs of fatty acids, which can subsequently be utilized in metabolic processes (Munir et al., 2019). Even though they were not selected as discriminatory, some changes showing a lower level in NF2mt samples were observed (Table S4). This observation is consistent with the report, which presents that elevated TG levels have been reported in meningiomas with higher malignancy (Safari Yazd et al., 2023). Thus, further studies based on detailed TG profiling are needed.

Lastly, medium- and long-chain acylcarnitines were altered in NF2-mutant meningiomas, reflecting increased lipid turnover and fatty acid oxidation, despite not being selected as discriminatory analytes (Table S2) (Melone et al., 2018). This may be linked to the absence of merlin, which can promote higher proliferative potential and energy demand. Cancerous cell proliferation is associated with increased energy consumption. This demand could be fulfilled through glucose metabolism, the Warburg effect, or fatty acid oxidation (Melone et al., 2018). Although it was observed that NF2mt cells are characterized by higher dependence on lipid metabolism (Stepanova et al., 2017). Increased fatty oxidation is associated with an enhanced carnitine shuttle system, resulting in higher acylcarnitine levels. Reports based on the analysis of acylcarnitine profile, including also short acyl-chain acylcarnitines, in meningioma meningothelial with different NF2 mutation status, showed that acylcarnitine levels were higher in NF2mt meningiomas than wildtype (Bogusiewicz et al., 2025). However, it should be noted that in lipidomic studies, the data were normalized to the whole lipidome, whereas acylcarnitines were analyzed as raw peak intensities. Normalization of lipidomic data enabled the analysis of lipid species as a fraction of the whole lipidome, allowing consideration of the particular lipid/group of lipids within the entire extracted lipidome (Nam et al., 2020, 2024; Sun & Xia, 2024). The lipidomic analysis presented herein provides a broader assessment, indicating that the overall lipidome undergoes significant alterations. Moreover, the profile of acylcarnitines presented herein is smaller due to limitations of LipidSearch software, enabling analysis of only medium and long-chain acylcarnitines being classified as fatty acid derivatives.

Finally, models built on all detected analytes provided better discrimination power of NF2 mutation status than those based on selected lipids alone. A combination of complementary chromatographic approaches (RPLC and HILIC) and ionization modes enhanced model performance by capturing both hydrophobic and hydrophilic lipid species. It could be related to the introduction of different information using different types of instrumental analysis. For instance, HILIC enables the separation of hydrophilic analytes such as phospholipids sphingomyelins, and RPLC, on the other hand, detects hydrophobic analytes such as glycerides and ceramides (Cajka & Fiehn, 2014, 2016). The majority of analytes are detected in positive ion mode. However, analytes such as PI, PA, PS, and fatty acids are more commonly observed in negative ionization mode (Ivanova et al., 2001). This shows the importance of wide lipidomic coverage to obtain a comprehensive view of tumor metabolism and improve prediction parameters.

## Conclusions

Among lipids altered by NF2 mutation were phospholipids, sphingolipids, and acylcarnitines. Interestingly, the trend of elevated levels of PC and PE plasmogenes and acylcarnitines was observed in NF2mt meningiomas. The best differentiation of NF2 mutant and wild-type samples was achieved using the full profile of analytes obtained through RPLC and HILIC analysis in positive ion mode. However, the elevation of the number of analytes revealed that the most effective predictive performance was provided by LC–MS in negative ionization mode, which enabled the best discrimination between NF2 wild-type and NF2-mutated meningiomas, with 87.5% sensitivity and 88.9% specificity. The same prediction outcome was obtained using only six selected Hn lipids and by the combination of Hn and Rn where 15 analytes were selected. If there is a need to limit the chromatographic methods to one, the discriminant model using all 16 or reduced lipids determined by Hn had the best sensitivity and specificity.

## Supplementary Information

Below is the link to the electronic supplementary material.

Supplementary Material 1 (PDF 766 kb)

## Data Availability

The experimental data that support the findings of this study are available in the 10.18150/R0ZNBL.

## Abbreviations

AUC: area under curve
Cer: Ceramide
ChE: cholesterol ester
CV: coefficient of variation
DIVA: discriminating variable test
HexCer: hexoylcramide
HILIC: Hydrophilic interaction chromatography
Hn: HILIC in negative mode
Hp: HILIC in positive mode
KLF4: Kruppel-like factor 4
LC-MS: Liquid chromatography coupled with mass spectrometry
LC-HRMS: Liquid chromatography coupled with high resolution mass spectrometry
LPC: Lysophosphatidylcholine
LPE: Lysophosphatidylethanolamine
MAD: median absolute deviation
MCCR: mean correct classification rate
MTBE: Methyl tert-butyl ether
NF2m: NF2 mutant
NF2w: NF2 wildtype
NPLC: normal phase chromatography
PA: Phosphatidic acid
PC: Phosphatidylcholine
PE: Phosphatidylethanolamine
PI: Phosphatidylinositol
PLS-DA: partial least squares
PS: Phosphatidylserine
QC: quality control
Rn: RPLC in negative mode
ROC: receiver operating characteristic
Rp: RPLC in positive mode
RPLC: Reversed-phase liquid chromatography
SM: Sphingomyelin
SPB: Sphingosine
SPME: solid-phase microextraction
SR: selectivity ratio
TP: target projection
TRAF7: tumor necrosis factor receptor-associated factor 7
WHO: World Health Organization
