# Supplementary material for: Lipidomic profile of meningiomas harboring different NF2 mutation status
Source: Metabolomics. 2026 May 7;22(3):68. doi: 10.1007/s11306-026-02399-4 (PMC13153021; doi:10.1007/s11306-026-02399-4)
Supplement: Supplementary file 1 — Supplementary Material 1 (PDF 766 kb) [file 11306_2026_2399_MOESM1_ESM.pdf]

Lipidomic profile of meningiomas harboring different NF2 mutation status

Joanna Bogusiewicz[1], Ivana Stanimirova[2], Magdalena Gaca-Tabaszewska[1], Paulina Szeliska[1], Krystyna Soszyńska[3], Anna Majdańska[3], Agata Ryfa[3], Alicja Bartoszewska-Kubiak[3], Jacek Furtak[4,5], Marcin Birski[5], Marek Harat[4,5*], Barbara Bojko[1*]

[1] Department of Pharmacodynamics and Molecular Pharmacology, Faculty of Pharmacy, Collegium Medicum in Bydgoszcz, Nicolaus Copernicus University in Torun, 85-089 Bydgoszcz, Poland

[2] Institute of Chemistry, University of Silesia in Katowice, 40-006 Katowice, Poland

[3] Laboratory of Clinical Genetics and Molecular Pathology, Department of Pathology, 10th Military Research Hospital and Polyclinic, Bydgoszcz, Poland, 85-681 Bydgoszcz, Poland

[4] Medical Faculty, Bydgoszcz University of Science and Technology, 85-796 Bydgoszcz, Poland

[5] Department of Neurosurgery, 10th Military Research Hospital and Polyclinic, 85-681 Bydgoszcz, Poland

Corresponding authors:

Professor Barbara Bojko, Department of Pharmacodynamics and Molecular Pharmacology, Faculty of Pharmacy, Collegium Medicum in Bydgoszcz, Nicolaus Copernicus University in Torun, Jurasza 2, 85-089 Bydgoszcz, Poland; bbojko@cm.umk.pl

Professor Marek Harat; Department of Neurosurgery, 10th Military Research Hospital and Polyclinic, Powstańców Warszawy 5, 85-681 Bydgoszcz, Poland; harat@10wsk.mil.pl

SUPPLEMENTARY MATERIALS

Genetic testing

Tumor specimens were formalin-fixed and paraffin-embedded. All samples were classified by histopathological examination and graded according to the WHO 2016 guidelines. DNA was extracted using the Maxwell 16 FFPE Plus LEV DNA Purification Kit and Maxwell 16 Instrument (Promega Corporation, Fitchburg, WI, USA). DNA samples were purified using the DNA Clean & Concentrator Kit (Zymo Research, Irvine, CA, USA). For multiplex ligation-dependent probe amplification (MLPA), DNA was isolated from the blood of healthy volunteers for use as controls.

MLPA and the SALSA MLPA P044-C1 kit (MRC-Holland, Amsterdam, the Netherlands) were used to detect loss (deletions) of the NF2 gene. MLPA assays were carried out by PCR according to the manufacturer's protocol using 50 ng of normal and tumor DNA. Reference samples were included in each experiment. The PCR, DNA denaturation, and ligation steps were performed according to the manufacturer's instructions. Amplified PCR products were separated by electrophoresis on an ABI PRISM 310 genetic analyzer (Thermo Fisher Scientific, Waltham, MA, USA) and, as an internal size standard, the LIZ-500 Genescan (Thermo Fisher Scientific) was used. Data were analyzed using the MRC-Coffalyser.Net (MRC-Holland).

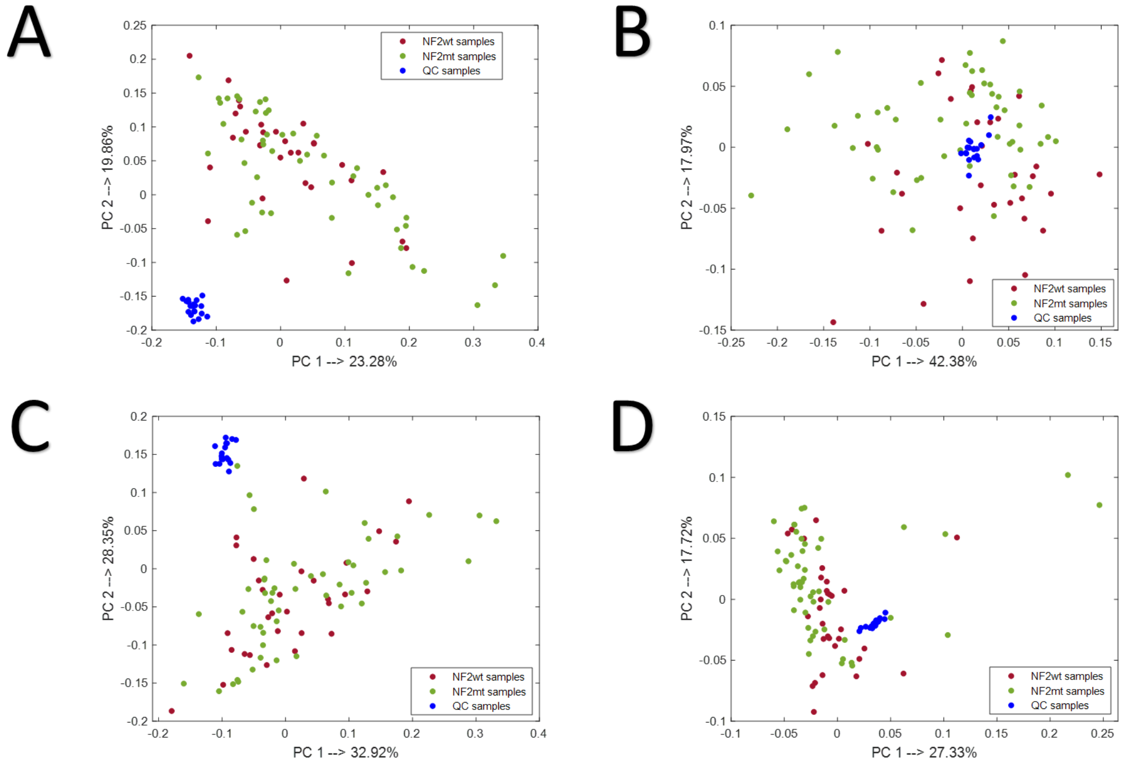

Figure S1 PCA visualizing QC for A. Hp, B. Hn, C. Rp, D. Rn. As it is visible, the coordinates on the plot represent a tight cluster

Table S1
Details on patients included in the study
AP – meningioma anaplasticum, AT – meningioma atypicum, F – female, M – male, MA – meningioma angiomatous, MM/MA – meningioma meningiotheliae – angiomatous, MF – meningioma fibrosum, MF/MP – meningioma fibrosum – psammomamatosum, MM – meningioma meningiotheliae, MM/MF – meningioma meningiotheliae – fibrosum, MM/MP – meningioma meningiotheliae – psammomamatosum, MP – meningioma psammomamatosum, mut – mutant, wt – wildtype

LP	AGE	SEX	HISTOLOGY	GRADE	NF
**8**	40	F	MF/MP	1	mt
**10**	58	F	MF	1	mt
**11**	59	F	MF	1	mt
**16**	30	F	MA	1	mt
**18**	79	F	MM	1	mt
**24**	56	F	MP	1	mt
**26**	65	F	MF	1	mt
**28**	56	F	MF	1	mt
**31**	51	F	MM/MP	1	mt
**33**	65	F	MF	1	mt
**36**	53	F	MP	1	mt
**43**	66	M	MP	1	mt
**52**	57	F	MP	1	mt
**56**	80	M	MM	1	mt
**57**	49	F	MM	1	mt
**59**	62	F	MP	1	mt
**65**	64	M	MM	1	mt
**67**	39	F	MM	1	mt
**68**	61	F	AT	2	mt
**69**	66	F	MP	1	mt
**70**	56	M	MM	1	mt
**75**	55	F	MF	1	mt
**82**	56	F	MF	1	mt
**95**	54	F	MP	1	mt
**98**	72	F	AP	3	mt
**103**	55	F	MM	1	mt
**105**	41	F	MM/MP	1	mt
**106**	70	F	MM/MP	1	mt
**109**	64	M	MM/MF	1	mt
**115**	76	F	MF	1	mt
**119**	80	F	MM/MF	1	mt
**126**	52	F	MM	1	mt
**134**	69	F	MM	1	mt
**140**	44	F	MM	1	mt
**142**	52	F	MM/MF	1	mt
**147**	59	F	MF/MP	1	mt
**148**	63	F	MM	1	mt
**152**	56	F	MF	1	mt
**153**	54	F	MF	1	mt
**167**	70	M	MM	1	mt
**169**	39	F	MM/MF	1	mt
**171**	74	F	MP	1	mt
**172**	62	F	MF/MP	1	mt
**173**	71	M	MM	1	mt
**174**	56	F	MM/MF	1	mt
**178**	69	M	MM/MF	1	mt
**184**	60	F	MF	1	mt
**192**	61	F	MF	1	mt
**198**	67	F	MF	1	mt

**201**	40	F	MM	1	mt
**5**	60	F	MM	1	wt
**9**	43	F	MM	1	wt
**15**	40	M	MM	1	wt
**23**	63	F	MM	1	wt
**29**	72	M	MM	1	wt
**32**	59	M	MF	1	wt
**34**	69	M	MM	1	wt
**53**	63	F	MM/MA	1	wt
**81**	52	F	MM	1	wt
**83**	66	M	MM/MP	1	wt
**84**	57	F	MP	1	wt
**85**	59	F	MM	1	wt
**89**	31	F	MM/MF	1	wt
**97**	44	F	MP	1	wt
**101**	59	F	MM	1	wt
**107**	58	M	MA	1	wt
**114**	65	F	MM	1	wt
**118**	48	M	MM	1	wt
**121**	43	M	AT	2	wt
**123**	43	F	MM	1	wt
**127**	67	F	MM	1	wt
**159**	40	F	MM	1	wt
**161**	39	F	MM	1	wt
**163**	54	F	MM	1	wt
**168**	59	F	MM	1	wt
**175**	41	M	MM	1	wt
**182**	73	F	MM	1	wt
**189**	74	F	MM	1	wt
**190**	69	M	MM/MP	1	wt
**195**	69	F	MM/MP	1	wt
**202**	45	F	MM	1	wt

Table S2 The list of lipid species detected using Hp.

Analyte	Adduct	m/z	RT	NF2wt	NF2mt	NF2mt: NF2wt	p-value
**AcCa 10:0**	$[M+H]^+$	316.2482	8.23	0.0055±0.0049	0.0087±0.0064	1.43	0.145
**AcCa 10:1**	[M+H]+	314.2326	8.30	0.0017±0.0017	0.0015±0.0012	0.86	0.586
**AcCa 12:0**	$[M+H]^+$	344.2795	7.99	0.0027±0.0018	0.0033±0.0021	1.27	0.105
**AcCa 12:1**	$[M+H]^+$	342.2639	8.02	0.0020±0.0014	0.0018±0.0013	0.97	0.420
**AcCa 14:0**	$[M+H]^+$	372.3108	7.80	0.0019±0.0013	0.0030±0.0016	1.52	0.016
**AcCa 14:1**	$[M+H]^+$	370.2952	7.78	0.0034±0.0026	0.0040±0.0024	1.12	0.206
**AcCa 16:0**	$[M+H]^+$	400.3421	7.65	0.0051±0.0019	0.0060±0.0025	1.52	0.074
**AcCa 16:1**	$[M+H]^+$	398.3265	7.66	0.0021±0.0015	0.0030±0.0016	1.35	0.109
**AcCa 18:1**	$[M+H]^+$	426.3578	7.53	0.0045±0.0022	0.0064±0.0033	1.32	0.221
**AcCa 18:2**	$[M+H]^+$	424.3421	7.59	0.0023±0.0016	0.0023±0.0014	1.10	0.547
**AcCa 8:0**	$[M+H]^+$	288.2169	8.59	0.0125±0.0109	0.0185±0.0147	1.48	0.080
**Cer 42:2;O2**	$[M+H]^+$	648.6289	1.60	$8.6.10^{-4}$±$1.7.10^{-4}$	$6.3.10^{-4}$±$1.8.10^{-4}$	0.95	0.001
**HexCer 36:1;O2**	$[M+H]^+$	728.6035	3.62	$9.9.10^{-6}$±$8.2.10^{-6}$	$1.0.10^{-5}$±$8.8.10^{-5}$	0.83	0.560
**HexCer 40:1;O3**	$[M+H]^+$	800.6610	5.39	$3.6.10^{-6}$±$1.9.10^{-6}$	$2.7.10^{-6}$±$1.3.10^{-6}$	1.18	0.120
**HexCer 41:1;O3**	$[M+H]^+$	814.6767	5.34	$4.3.10^{-5}$±$3.6.10^{-5}$	$1.9.10^{-5}$±$1.8.10^{-5}$	1.05	0.244
**HexCer 42:1;O3**	$[M+H]^+$	828.6923	5.29	$6.9.10^{-5}$±$6.7.10^{-5}$	$5.3.10^{-5}$±$5.1.10^{-5}$	0.90	0.676

**HexCer 42:2;O2**	$[M+H]^+$	810.6817	3.33	$3.1.10^{-4}\pm2.7.10^{-4}$	$4.3.10^{-4}\pm3.9.10^{-4}$	1.23	0.854
**HexCer 42:2;O3**	$[M+H]^+$	826.6767	5.35	$3.7.10^{-5}\pm2.8.10^{-5}$	$2.1.10^{-5}\pm1.4.10^{-5}$	1.03	0.029
**HexCer 43:1;O3**	$[M+H]^+$	842.7080	5.25	$9.8.10^{-7}\pm6.1.10^{-7}$	$5.7.10^{-7}\pm3.0.10^{-7}$	0.85	0.031
**HexCer 43:2;O2**	$[M+H]^+$	824.6974	3.28	$2.0.10^{-6}\pm1.5.10^{-6}$	$1.8.10^{-6}\pm1.2.10^{-6}$	1.25	0.346
**HexCer 44:2:O2**	$[M+H]^+$	838.7130	3.23	$6.2.10^{-6}\pm5.5.10^{-6}$	$3.5.10^{-6}\pm2.9.10^{-6}$	0.83	0.256
**HexCer 44:4;O2**	$[M+H]^+$	834.6817	3.23	$1.510^{-5}\pm1.4.10^{-5}$	$2.0.10^{-4}\pm2.0.10^{-4}$	1.66	0.069
**LPC 16:0***	$[M+H]^+$	496.3398	8.93	0.0088±0.0037	0.0049±0.0021	0.70	0.001
**LPC 18:0***	$[M+H]^+$	524.3711	8.77	0.0042±0.0012	0.0022±0.0009	0.78	0.002
**LPC 18:1**	$[M+H]^+$	522.3554	8.81	0.0025±0.0009	0.0017±0.0007	0.82	0.008
**LPC 20:3**	$[M+H]^+$	546.3554	8.78	0.0024±0.0009	0.0015±0.0006	0.66	<0.001
**LPC 20:4**	$[M+H]^+$	544.3398	8.46	0.0020±0.0004	0.0016±0.0004	0.94	0.019
**PC 30:0**	$[M+Na]^+$	728.5201	6.76	0.0036±0.0010	0.0033±0.0007	1.00	0.907
**PC P–32:1**	$[M+H]^+$	716.5589	7.42	$8.1.10^{-4}\pm2.3.10^{-4}$	$9.9.10^{-4}\pm2.6.10^{-4}$	1.25	0.019
**PC 34:1***	$[M+H]^+$	760.5851	6.54	0.1681±0.0332	0.1735±0.0185	1.79	0.200
**PC 34:2***	$[M+H]^+$	758.5694	6.56	0.0482±0.0126	0.0353±0.0104	0.72	<0.001
**PC 36:1***	$[M+H]^+$	788.6164	6.45	0.0312±0.0048	0.0354±0.0086	1.08	0.317
**PC 36:2***	$[M+H]^+$	786.6007	6.47	0.0369±0.0063	0.0284±0.0062	0.84	0.002
**PC 36:3**	$[M+Na]^+$	806.5670	6.46	0.0138±0.0026	0.0120±0.0038	0.88	0.120
**PC 36:4***	$[M+H]^+$	782.5694	6.46	0.1013±0.0111	0.1032±0.0118	0.98	0.683
**PC P–36:4**	$[M+H]^+$	766.5745	7.27	$2.8.10^{-4}\pm0.7.10^{-4}$	$2.8.10^{-4}\pm1.0.10^{-4}$	0.89	0.392
**PC 38:4***	$[M+H]^+$	810.6007	6.40	0.0475±0.0098	0.0443±0.0081	0.98	0.869
**PC 38:5***	$[M+H]^+$	808.5851	6.41	0.0281±0.0044	0.0234±0.0061	0.84	0.016
**PC 38:6***	$[M+H]^+$	806.5694	6.42	0.0134±0.0028	0.0117±0.0037	0.89	0.168
**PE 34:1***	$[M+H]^+$	718.5381	7.60	0.0080±0.0022	0.0077±0.0012	1.05	0.392
**PE O–34:1**	$[M+Na]^+$	726.5408	7.38	0.0034±0.0005	0.0036±0.0007	0.95	0.915
**PE P–34:1***	$[M+H]^+$	702.5432	7.45	0.0162±0.0059	0.0239±0.0052	1.37	0.001
**PE 36:1***	$[M+H]^+$	746.5694	7.43	0.0091±0.0020	0.0083±0.0013	0.94	0.403
**PE P–36:1***	$[M+H]^+$	730.5745	7.39	0.0063±0.0017	0.0057±0.0013	0.92	0.620
**PE 36:2***	$[M+H]^+$	744.5538	7.53	0.0049±0.0013	0.0036±0.0008	0.80	0.015
**PE P–36:2***	$[M+H]^+$	728.5589	7.40	0.0037±0.0012	0.0036±0.0007	0.86	0.371
**PE 36:3**	$[M+H]^+$	742.5381	7.45	0.0014±0.0003	0.0014±0.0003	0.85	0.037
**PE P–36:3**	$[M+H]^+$	726.5432	7.39	0.0029±0.0005	0.0029±0.0008	0.94	0.662
**PE P–36:4***	$[M+H]^+$	724.5276	7.36	0.0192±0.0065	0.0244±0.0070	1.16	0.064
**PE 38:2***	$[M+H]^+$	772.5851	7.36	$6.6.10^{-4}\pm2.0.10^{-4}$	$4.5.10^{-4}\pm1.2.10^{-4}$	0.71	0.002
**PE 38:3**	$[M+Na]^+$	792.5514	7.34	0.0030±0.0007	0.0034±0.0009	0.97	0.414
**PE P–38:4***	$[M+H]^+$	752.5589	7.27	0.0272±0.0065	0.0230±0.0039	0.75	0.001
**PE 38:5***	$[M+H]^+$	766.5381	7.43	0.0065±0.0016	0.0055±0.0012	0.81	0.031
**PE P–38:5**	$[M+H]^+$	750.5432	7.32	0.0126±0.0038	0.0114±0.0030	0.82	0.056
**PE 38:6***	$[M+H]^+$	764.5225	7.44	0.0024±0.0005	0.0022±0.0005	0.92	0.298
**PE P–38:6***	$[M+H]^+$	748.5276	7.29	0.0032±0.0007	0.0059±0.0021	1.61	<0.001
**PE P–40:4***	$[M+H]^+$	780.5902	7.23	0.0020±0.0005	0.0021±0.0007	0.93	0.800
**PE P- 40:5***	$[M+H]^+$	778.5745	7.25	0.0022±0.0005	0.0026±0.0009	1.15	0.077
**PE 40:6***	$[M+H]^+$	792.5538	7.36	0.0029±0.0007	0.0032±0.0008	0.97	0.341
**PE P-40:6***	$[M+H]^+$	776.5589	7.21	0.0035±0.0011	0.0048±0.0015	1.14	0.020
**PE 40:7***	$[M+H]^+$	790.5381	7.38	0.0114±0.0019	0.0111±0.0018	0.95	0.448
**PS 36:1***	$[M+H]^+$	790.5593	8.73	0.0105±0.0016	0.0101±0.0026	1.01	0.954

**PS 38:4***	$[M+H]^+$	812.5436	8.67	0.0145±0.0014	0.0144±0.0036	0.88	0.443
**SM 34:1;O2**	$[M+H]^+$	703.5749	8.09	0.0482±0.0124	0.0524±0.0119	1.06	0.240
**SM 36:4;O2**	$[M+H]^+$	725.5592	8.09	0.0286±0.0040	0.0328±0.0059	1.02	0.521
**SM 38:4;O2**	$[M+H]^+$	753.5905	8.01	0.0128±0.0065	0.0135±0.0051	0.86	0.690
**SM 38:5;O2**	$[M+H]^+$	751.5749	8.01	0.0011±0.0004	$9.5.10^{-4}$±$2.9.10^{-4}$	0.79	0.080
**SM 40:4;O2**	$[M+H]^+$	781.6218	7.91	0.0044±0.0011	0.0041±0.0012	0.89	0.156
**SM 44:4;O2**	$[M+H]^+$	837.6844	7.79	0.0123±0.0037	0.0136±0.0046	1.07	0.515
**SM 44:5;O2**	$[M+H]^+$	835.6688	7.81	0.0301±0.0068	0.0273±0.0079	0.91	0.203
**SPB 14:0;O2**	$[M+H]^+$	246.2428	11.57	$2.6.10^{-4}$±$1.6.10^{-4}$	$2.4.10^{-4}$±$1.3.10^{-4}$	0.72	0.892
**SPB 14:0;O2**	$[M+H]^+$	246.2428	11.87	$1.0.10^{-4}$±$0.7.10^{-4}$	$1.4.10^{-4}$±$0.9.10^{-4}$	1.00	0.648
**SPB 14:0;O3**	$[M+H]^+$	262.2377	3.70	0.0082±0.0042	0.0056±0.0040	0.88	0.923
**SPB 22:1;O3**	$[M+H]^+$	372.3472	4.98	0.0185±0.029	0.0178±0.0091	0.81	0.983

*sodium adduct was also identified

Table S3 The list of lipid species detected using Hn

Analyte	Adduct	m/z	RT	NF2wt	NF2mt	NF2mt: NF2wt	p-value
**PE 34:1**	$[M-H]^-$	716.5236	7.58	0.0407±0.0102	0.0453±0.0089	1.07	0.135
**PE P–34:1**	$[M-H]^-$	700.5287	7.44	0.0848±0.0362	0.1326±0.0283	1.44	<0.001
**PE 36:1**	$[M-H]^-$	744.5549	7.51	0.0473±0.0086	0.0461±0.0088	0.92	0.496
**PE P–36:1**	$[M-H]^-$	728.5600	7.36	0.0343±0.0100	0.0325±0.0076	0.96	0.634
**PE 36:2**	$[M-H]^-$	742.5392	7.51	0.0220±0.0063	0.0193±0.0038	0.82	0.018
**PE P–36:2**	$[M-H]^-$	726.5443	7.37	0.0183±0.0054	0.0176±0.0016	0.88	0.361
**PE P–36:4**	$[M-H]^-$	722.5130	7.30	0.0981±0.0205	0.0866±0.0197	0.90	0.190
**PE 38:4**	$[M-H]^-$	766.5392	7.37	0.0879±0.0174	0.0891±0.0138	1.06	0.236
**PE P–38:4**	$[M-H]^-$	750.5443	7.24	0.1129±0.0325	0.0869±0.0175	0.72	0.001
**PE P–38:5**	$[M-H]^-$	748.5287	7.24	0.0433±0.0112	0.0417±0.0109	0.86	0.366
**PE 38:6**	$[M-H]^-$	762.5079	7.42	0.0043±0.0020	0.0068±0.0021	1.20	0.032
**PE P–38:6**	$[M-H]^-$	746.5130	7.27	0.0108±0.0038	0.0253±0.0073	2.16	<0.001
**PE 40:6**	$[M-H]^-$	790.5392	7.33	0.0039±0.0018	0.0072±0.0019	1.10	<0.001
**PE P–40:6**	$[M-H]^-$	774.5443	7.19	0.0081±0.0032	0.0158±0.0044	1.45	<0.001
**PI 38:4**	$[M-H]^-$	885.5499	7.31	0.2043±0.0265	0.2018±0.0279	1.01	0.683
**PS 36:1**	$[M-H]^-$	788.5447	8.61	0.1463±0.0322	0.1313±0.0297	0.93	0.294

Table S4 The list of lipid species detected using Rp.

Analyte	Adduct	m/z	RT	NF2wt	NF2mt	NF2mt: NF2wt	p-value
**Cer 42:2;O2**	$[M+H]^+$	648.6289	11.36	0.0029±0.0018	0.0021±0.0012	1.98	0.586
**HexCer 36:1;O2**	$[M+H]^+$	728.6035	9.25	$7.6.10^{-5}$±$7.5.10^{-5}$	$5.4.10^{-5}$±$4.2.10^{-5}$	0.34	0.496
**HexCer 42:1;O2**	$[M+H]^+$	812.6974	11.26	$5.4.10^{-4}$±$2.1.10^{-4}$	$8.7.10^{-4}$±$4.0.10^{-4}$	1.26	0.013
**HexCer 42:1;O3**	$[M+H]^+$	828.6923	11.10	$1.3.10^{-4}$±$1.1.10^{-4}$	$9.2.10^{-5}$±$8.1.10^{-5}$	0.94	0.698
**HexCer 42:2;O2**	$[M+H]^+$	810.6817	10.76	$5.0.10^{-4}$±$3.4.10^{-4}$	$5.9.10^{-4}$±$4.1.10^{-4}$	1.37	0.145
**HexCer 42:2;O3**	$[M+H]^+$	826.6767	10.58	$1.8.10^{-5}$±$1.8.10^{-5}$	$1.1.10^{-5}$±$1.2.10^{-5}$	1.65	0.333
**HexCer 43:1;O3**	$[M+H]^+$	842.7080	11.36	$1.3.10^{-4}$±$1.1.10^{-4}$	$2.7.10^{-4}$±$2.4.10^{-4}$	1.19	0.749
**HexCer 43:2;O3**	$[M+H]^+$	840.6923	10.87	$4.2.10^{-5}$±$2.9.10^{-5}$	$3.7.10^{-5}$±$2.9.10^{-5}$	1.13	0.203

**HexCer 44:2;O2**	$[M+H]^+$	838.7130	11.29	$9.7.10^{-5}±8.7.10^{-5}$	$3.2.10^{-5}±2.4.10^{-5}$	0.47	0.089
**ChE 18:2**	$[M+NH4]^+$	666.6184	13.87	0.0258±0.0113	0.0149±0.0121	1.01	0.331
**ChE 20:4**	$[M+NH4]^+$	690.6184	13.79	0.0053±0.0040	0.0024±0.0020	2.27	0.326
**PC 32:1**	$[M+Na]^+$	754.5357	8.50	0.0028±0.0017	0.0032±0.0015	1.01	0.808
**PC 34:0**	$[M+Na]^+$	784.5827	9.88	0.0014±0.0007	0.0017±0.0009	0.97	0.377
**PC 34:1***	$[M+H]^+$	760.5851	9.31	0.0380 ±0.0223	0.0449±0.0158	1.08	0.534
**PC 36:3**	$[M+Na]^+$	806.5670	9.09	0.0078±0.0051	0.0070±0.0040	0.87	0.409
**PC 38:5**	$[M+H]^+$	808.5851	9.62	0.0032±0.0021	0.0023±0.0014	0.88	0.420
**PE 34:1**	$[M+H]^+$	718.5381	9.44	0.0124±0.0069	0.0125±0.0069	1.04	0.831
**PE P–34:1***	$[M+H]^+$	702.5432	9.86	0.0279±0.0146	0.0462±0.0223	1.36	0.020
**PE 36:1***	$[M+H]^+$	746.5694	10.18	0.0192±0.012	0.0164±0.0099	0.95	0.938
**PE 36:2***	$[M+H]^+$	744.5538	9.62	0.0075±0.0052	0.0057±0.0032	0.77	0.346
**PE P–36:2***	$[M+H]^+$	728.5589	10.00	0.0076±0.0054	0.0079±0.0050	0.78	0.938
**PE P–36:4***	$[M+H]^+$	724.5276	9.39	0.0294±0.0177	0.0209±0.0133	0.88	0.669
**PE P–38:3***	$[M+H]^+$	754.5745	10.19	0.0022±0.0011	0.0016±0.0007	1.01	0.726
**PE P–38:4***	$[M+H]^+$	752.5589	9.97	0.0054±0.0040	0.0061±0.0044	0.83	0.547
**PE 38:5***	$[M+H]^+$	766.5381	9.14	0.0028±0.0023	0.0024±0.0018	0.80	0.366
**PE P–38:5**	$[M+H]^+$	750.5432	9.55	0.0122±0.0086	0.0087±0.0066	0.70	0.156
**PE P-38:6**	$[M+H]^+$	748.5276	9.33	0.0027±0.0018	0.0061±0.0038	1.82	0.007
**PE P–40:5**	$[M+H]^+$	778.5745	10.10	$6.0.10^{-4}±5.9.10^{-4}$	$9.6.10^{-4}±7.7.10^{-4}$	1.27	0.322
**PE 40:6**	$[M+H]^+$	792.5538	9.71	0.0012±0.0008	0.0023±0.0014	0.71	0.009
**PE P–40:6**	$[M+H]^+$	776.5589	10.08	0.0022±0.0015	0.0045±0.0028	1.10	0.019
**PI 38:4**	$[M+NH4]^+$	904.5910	8.06	0.0109±0.0063	0.0104±0.0053	0.96	0.946
**PS 36:1***	$[M+H]^+$	790.5593	8.60	0.0215±0.0117	0.0261±0.0112	1.03	0.662
**PS 38:4***	$[M+H]^+$	812.5436	8.14	0.0025±0.0016	0.0026±0.0017	1.31	0.193
**PS 40:6**	$[M+H]^+$	836.5436	8.11	0.7322±0.5935	0.0017±0.0012	0.65	0.013
**SM 36:4;O2**	$[M+H]^+$	725.5592	8.10	0.0083±0.0034	0.0115±0.0049	1.16	0.272
**SPB 16:0;O3**	$[M+H]^+$	290.2690	3.17	0.0504±0.0282	0.0600±0.0296	1.06	0.627
**SPB 22:1;O3**	$[M+H]^+$	372.3472	3.31	0.0061±0.0027	0.0070±0.0026	0.90	0.915
**AC C16:0**	$[M+H]^+$	400.3421	3.78	0.0038±0.0011	0.0052±0.0024	1.42	0.224
**DG 32:0**	$[M+NH_4]^+$	586.5405	10.72	0.0175±0.0046	0.0142±0.0055	0.97	0.540
**DG 32:1**	$[M+NH_4]^+$	584.5248	10.27	0.0177±0.0062	0.0112±0.0064	0.91	0.079
**DG 34:0**	$[M+NH_4]^+$	614.5718	11.32	0.0099±0.0037	0.0097±0.0041	0.99	0.992
**DG 34:1**	$[M+NH_4]^+$	612.5562	10.86	0.0225±0.0072	0.0218±0.0067	1.12	0.892
**DG 36:1**	$[M+NH_4]^+$	640.5874	11.42	0.0050±0.0023	0.0055±0.0018	1.39	0.698
**MG 16:0**	$[M+NH_4]^+$	348.3108	4.73	0.0769±0.0443	0.0645±0.0557	1.23	0.801
**TG 44:2**	$[M+NH_4]^+$	764.6763	12.76	0.0033±0.0015	0.0023±0.0012	0.85	0.228
**TG 48:1**	$[M+NH_4]^+$	822.7545	13.61	0.0613±0.0133	0.0417±0.0183	0.83	0.052
**TG 50:1***	$[M+NH_4]^+$	850.7858	13.86	0.0202±0.0070	0.0171±0.0066	0.95	0.521
**TG 50:2***	$[M+NH_4]^+$	848.7702	13.66	0.0546±0.0207	0.0503±0.0196	0.89	0.377
**TG 50:3**	$[M+NH_4]^+$	846.7545	13.45	0.0195±0.0052	0.0143±0.0062	0.77	0.046
**TG 51:2**	$[M+NH_4]^+$	862.7858	13.79	0.0187±0.0040	0.0118±0.0051	0.76	0.014
**TG 52:1**	$[M+NH_4]^+$	878.8171	14.12	0.0247±0.0096	0.0206±0.0089	0.92	0.509
**TG 52:2***	$[M+NH_4]^+$	876.8015	13.90	0.0637±0.0295	0.0663±0.0351	0.92	0.808
**TG 52:3***	$[M+NH_4]^+$	874.7858	13.72	0.0392±0.0175	0.0320±0.0166	0.84	0.331
**TG 52:4**	$[M+NH_4]^+$	872.7702	13.52	0.0146±0.0070	0.0122±0.0075	1.00	0.317

**TG 54:0**	[M+NH$_4$]$^+$	908.8641	14.50	0.0037±0.0015	0.0028±0.0016	0.72	0.058
**TG 54:3***	[M+NH$_4$]$^+$	902.8171	13.96	0.0379±0.0182	0.0310±0.0126	0.98	0.312

*sodium adduct was also identified

Table S5 The list of lipid species detected using Rn

Analyte	Adduct	m/z	RT	NF2wt	NF2mt	NF2mt: NF2wt	p-value
**Cer 34:1;O2**	[M+CH3COO]$^-$	596.5259	9.21	0.0186±0.0044	0.0182±0.0070	1.31	0.854
**Cer 36:1;O2**	[M+CH3COO]$^-$	624.5572	10.02	0.0134±0.0038	0.0081±0.0029	0.87	0.032
**Cer 42:2;O2**	[M+CH3COO]$^-$	706.6355	11.35	0.0405±0.0066	0.0299±0.0077	1.03	0.022
**PC 32:1**	[M+CH3COO]$^-$	790.5604	8.51	0.0158±0.0034	0.0168±0.0025	1.03	0.613
**PC 34:1**	[M+CH3COO]$^-$	818.5917	9.29	0.1902±0.0202	0.1984±0.0164	1.04	0.165
**PC 36:1**	[M+CH3COO]$^-$	846.6230	10.04	0.0550±0.0056	0.0594±0.0110	1.07	0.168
**PC 36:2**	[M+CH3COO]$^-$	844.6073	9.51	0.0400±0.0106	0.0324±0.0083	0.82	0.026
**PE 34:1**	[M-H]$^-$	716.5236	9.42	0.0268±0.0059	0.0284±0.0040	0.97	0.977
**PE P–34:1**	[M-H]$^-$	700.5287	9.85	0.0790±0.0247	0.1123±0.0233	1.31	0.002
**PE 36:1**	[M-H]$^-$	744.5549	10.16	0.0518±0.0098	0.0458±0.0052	0.89	0.044
**PE P–36:1**	[M-H]$^-$	728.5600	10.51	0.0413±0.0169	0.0440±0.0086	0.88	0.437
**PE 36:2**	[M-H]$^-$	742.5392	9.60	0.0172±0.0050	0.0130±0.0047	0.76	0.007
**PE P–36:2**	[M-H]$^-$	726.5443	9.98	0.0216±0.0063	0.0206±0.0048	0.85	0.264
**PE P–36:4**	[M-H]$^-$	722.5130	9.37	0.0660±0.0237	0.0654±0.0204	0.87	0.303
**PE P–38:4**	[M-H]$^-$	750.5443	9.95	0.0204±0.0081	0.0207±0.0081	0.72	0.540
**PE 40:4**	[M-H]$^-$	794.5705	10.26	0.0039±0.0029	0.0034±0.0017	0.72	0.969
**PE 40:6**	[M-H]$^-$	790.5392	9.69	0.0019±0.0014	0.0044±0.0018	0.90	0.001
**PE P–40:6**	[M-H]$^-$	774.5443	10.06	0.0063±0.0040	0.0132±0.0048	1.44	<0.001
**PI 38:4**	[M-H]$^-$	885.5499	8.02	0.0471±0.0084	0.0450±0.0095	0.97	0.876
**PS 36:1**	[M-H]$^-$	788.5447	8.55	0.0588±0.0097	0.0562±0.0098	0.95	0.504
**PS 40:6**	[M-H]$^-$	834.5291	8.04	0.0011±0.0008	0.0022±0.0012	0.90	0.003
**SM 34:1;O2**	[M+CH3COO]$^-$	761.5814	8.08	0.0481±0.0078	0.0558±0.0072	1.05	0.206
**SM 36:1;O2**	[M+CH3COO]$^-$	789.6127	8.99	0.0230±0.0071	0.0251±0.0063	0.92	0.734
**SM 40:1;O2**	[M+CH3COO]$^-$	845.6753	10.50	0.0390±0.0065	0.0447±0.0111	1.13	0.125

Table S6 Descriptive statistics for the metabolites that were found to be most important using the PLS-DA with the target projection approach. The median values ± MAD (median absolute deviation) of the pairwise models are only listed for those metabolites that are important.

**Metabolites**	**Median contents of metabolites ±MAD**		**Kruskal-Wallis test**
	**NF2wt**	**NF2mt**	**(p-value)**
**Hp**			
HexCer 40:1, O3	$3.6.10^{-6}\pm1.9.10^{-6}$	$2.7.10^{-6}\pm1.3.10^{-6}$	0.120
HexCer 42:1, O3	$6.9.10^{-5}\pm6.7.10^{-5}$	$5.3.10^{-5}\pm5.1.10^{-5}$	0.676
HexCer 42:2, O3	$3.7.10^{-5}\pm2.8.10^{-5}$	$2.1.10^{-5}\pm1.4.10^{-5}$	0.029
HexCer 43:1, O3	$9.8.10^{-7}\pm6.1.10^{-7}$	$5.7.10^{-7}\pm3.0.10^{-7}$	0.031
HexCer 43:2, O2	$2.0.10^{-6}\pm1.5.10^{-6}$	$1.8.10^{-6}\pm1.2.10^{-6}$	0.346
HexCer 44:2, O2	$6.2.10^{-6}\pm5.5.10^{-6}$	$3.5.10^{-6}\pm 2.9.10^{-6}$	0.256
PC P-36:4	$2.8.10^{-4}\pm0.7.10^{-4}$	$2.8.10^{-4}\pm1.0.10^{-4}$	0.392
PE P-38:4	0.0272±0.0065	0.0230 ± 0.0039	0.001
**Hn**			
PE P 36:2	0.0220 ± 0.0063	0.0193 ± 0.0038	0.018
PE P 36:4	0.0981 ± 0.0205	0.0866 ± 0.0197	0.190
PE 38:6	0.0043 ± 0.0020	0.0068 ± 0.0021	0.032
PEP 38:6	0.0108 ± 0.0038	0.0253 ± 0.0073	<0.001
PE 40:6	0.0039 ± 0.0018	0.0072 ± 0.0019	<0.001
PEP 40:6	0.0081 ± 0.0032	0.0158 ± 0.0044	<0.001
**Rp**			
HexCer 42:1,O2	$5.4.10^{-4}\pm 2.1.10^{-4}$	$8.7.10^{-4}\pm 4.0.10^{-4}$	0.013
HexCer 42:2,O2	$5.0.10^{-4}\pm 3.4.10^{-4}$	$5.9.10^{-4}\pm 4.1.10^{-4}$	0.145
HexCer 42:2,O3	$1.8.10^{-5}\pm 1.8.10^{-5}$	$1.1.10^{-5}\pm 1.2.10^{-5}$	0.333
HexCer 43:1,O3	$1.3.10^{-4}\pm 1.1.10^{-4}$	$2.7.10^{-4}\pm 2.4.10^{-4}$	0.749
HexCer 43:2,O3	$4.2.10^{-5}\pm 2.9.10^{-5}$	$3.7.10^{-5}\pm 2.9.10^{-5}$	0.203
HexCer 44:2,O2	$9.7.10^{-5}\pm 8.7.10^{-5}$	$3.2.10^{-5}\pm 2.4.10^{-5}$	0.089
PC 34:0	0.0014 ± 30.0007	0.0017 ± 0.0009	0.377
PE P-40:5	$6.0.10^{-4}\pm 5.9.10^{-4}$	$9.6.10^{-4}\pm 7.7.10^{-4}$	0.322
PE 40:6	0.0012 ± 0.0008	0.0023 ± 0.0014	0.009
PE P-40:6	0.0022 ± 0.0015	0.0045 ±0.0028	0.019
PS 40:6	0.7322 ±0.5935	0.0017 ± 0.0012	0.013
**Rn**			
PC 32:1	0.0158 ± 0.0034	0.0168 ± 0.0025	0.613
PC 36:1	0.0550 ± 0.0056	0.0594 ± 0.0110	0.168
PE 34:1	0.0268 ± 0.0059	0.0284 ± 0.0040	0.977
PE P-36:1	0.0113 ± 0.0169	0.0440 ± 0.0086	0.437
PE 36:2	0.0172 ± 0.0050	0.0130 ± 0.0047	0.007
PE 40:4	0.0039 ± 0.0029	0.0034 ± 0.0017	0.969
PE 40:6	0.0019 ± 0.0014	0.0044 ± 0.0018	0.001
PS 40:6	0.0011 ± 0.0008	0.0022 ± 0.0012	0.003
SM 36:1, O2	0.0230 ± 0.0071	0.0251 ± 0.0063	0.734

Table S7. Fragmentation pattern for lipids with rare occurrence in biological samples – PC 16:0/20:3 + Na+ in Rp analysis: m-score: 49.2; T-score: 0.3; Occupancy 80.1

OBSMZ	TYPE	IT.(%)	FRAG.	DELTA(DA)
57.0708	MS2	5.475		
59.0612	MS2	9.613		
62.9825	MS2	2.153		
64.9783	MS2	1.771		
67.055	MS2	5.505		
69.0706	MS2	3.9		
71.0736	MS2	2.24		
71.0862	MS2	5.753	C5H11	0.0007
79.0549	MS2	2.778	C6H7	0.0007
81.0705	MS2	7.148	C6H9	0.0006
83.0861	MS2	4.305	C6H11	0.0006
85.1018	MS2	3.876	C6H13	0.0006
86.097	MS2	54.363	C5H12N	0.0006
87.1004	MS2	1.798	C5H12N [isotope]	1.0039
89.0603	MS2	1.705		
93.0704	MS2	5.594	C7H9	0.0005
95.086	MS2	7.784	C7H11	0.0005
97.1017	MS2	2.575	C7H13	0.0005
104.1073	MS2	1.806		
104.9927	MS2	1.738		
105.0701	MS2	1.014		
107.0861	MS2	2.754	C8H11	0.0005
109.1017	MS2	3.703	C8H13	0.0005
121.1013	MS2	4.061	C9H13	0.0001
123.1172	MS2	1.082	C9H15	0.0004
125.0001	MS2	2.592		
126.0893	MS2	1.136		
128.9537	MS2	8.428		
133.1013	MS2	1.228		
135.1169	MS2	1.257	C10H15	0.0001
146.9819	MS2	100	C2H5O4NaP	0.0002
147.9852	MS2	1.531	C2H5O4NaP [isotope]	1.0034
163.1478	MS2	1.645		
184.0736	MS2	24.355		
188.0083	MS2	4.634		
253.0916	MS2	1.033		
311.2935	MS2	1.027		
437.2054	MS2	2.362		
478.3294	MS2	1.476	LPC(16:0)-OH	0.0002
491.2556	MS2	3.148	M+Na-N(CH3)3-FA(16:0)	0.0023
601.5203	MS2	10.971	NL[PC,+Na]+H	0.0013
602.5233	MS2	4.673	NL[PC,+Na]+H [isotope]	1.0042
623.5022	MS2	42.833	NL[PC]	0.0012
624.5052	MS2	21.155	NL[PC] [isotope]	1.0043
747.4938	MS2	31.298	M+Na-N(CH3)3	0.0002
748.4979	MS2	16.984	M+Na-N(CH3)3 [isotope]	1.0043
806.5671	MS2	41.312	M+Na	0.0001
807.5704	MS2	23.114		

Table S8. Fragmentation pattern for lipids with rare occurrence in biological samples – HexCer 43:1;O3 +H+ in Rp analysis: M-score: 47.5; T-score: 1; Occupancy: 79.1

OBSMZ	TYPE	IT.(%)	FRAG.	DELTA(DA)
60.0452	MS2	1.789		
67.055	MS2	1.759		
69.0706	MS2	1.37		
71.0862	MS2	1.075		
81.0705	MS2	2.831		
82.0656	MS2	2.146		
83.0861	MS2	1.475		
86.097	MS2	1.038		
93.0703	MS2	1.747		
95.086	MS2	2.685		
97.1016	MS2	1.789		
107.0859	MS2	1.207		
109.1015	MS2	1.118		
121.1014	MS2	2.552		
135.1169	MS2	1.703		
184.0734	MS2	12.257		
247.2424	MS2	1.004		
252.2686	MS2	17.715	So(d18:1)-H2O-CH2O	0
253.272	MS2	2.669	So(d18:1)-H2O-CH2O [isotope]	1.0034
264.2687	MS2	100	So(d18:1)-2H2O	0.0001
265.2721	MS2	15.208	So(d18:1)-2H2O [isotope]	1.0035
282.2794	MS2	7.66	So(d18:1)-H2O	0.0003
283.2829	MS2	1.29	So(d18:1)-H2O [isotope]	1.0037
398.3995	MS2	2.127		
632.6342	MS2	4.044		
633.6369	MS2	1.719		
644.634	MS2	17.368	NL[G1,H2O]	0
645.6375	MS2	8.641	NL[G1,H2O] [isotope]	1.0035
646.649	MS2	2.038		
647.6542	MS2	1.257		
662.6447	MS2	10.435	NL[G1]	0.0001
663.6481	MS2	4.807	NL[G1] [isotope]	1.0035
824.6962	MS2	3.723	NL[H2O]	-0.0012
825.7001	MS2	2.56	NL[H2O] [isotope]	1.0027

Table S9. Fragmentation pattern for lipids with rare occurrence in biological samples – HexCer 43:2;O3 +H+ in Rp analysis; M-score: 9.1; T-score: 1.2; Occupancy: 22.7

OBSMZ	TYPE	IT.(%)	FRAG.	DELTA(DA)
59.0612	MS2	1.872		
60.0452	MS2	1.244		
67.055	MS2	2.437		
69.0706	MS2	1.404		
71.0862	MS2	1.581		
79.0548	MS2	1.481		
81.0705	MS2	3.298		
82.0656	MS2	2.045		
83.0861	MS2	1.516		
85.1016	MS2	1.102		

95.086	MS2	3.904		
97.1017	MS2	1.692		
109.1015	MS2	2.461		
121.1015	MS2	1.582		
128.9536	MS2	1.429		
135.1168	MS2	1.77		
184.0734	MS2	5.554		
247.2419	MS2	1.404		
252.2686	MS2	16.362		
253.2721	MS2	2.279		
264.2687	MS2	100		
265.2722	MS2	16.025		
266.2845	MS2	1.923		
282.2792	MS2	8.139	So(d18:0+pO)-2H2O	0.0001
630.6197	MS2	3.81		
631.6202	MS2	1.301		
642.6176	MS2	16.503	NL[G1,H2O]	-0.0007
643.622	MS2	8.849	NL[G1,H2O] [isotope]	1.0037
644.6335	MS2	2.211		
660.6285	MS2	8.821	NL[G1]	-0.0004
661.6326	MS2	5.117	NL[G1] [isotope]	1.0037
822.6812	MS2	4.229	NL[H2O]	-0.0005
823.6857	MS2	2.137	NL[H2O] [isotope]	1.004
839.6674	MS2	1.604		

Table S10. Fragmentation pattern for lipids with rare occurrence in biological samples – PC 30:0+Na+ in Hp analysis; M-score: 48.4; T-score: 0.1; Occupancy: 67.8

OBSMZ	TYPE	IT.(%)	FRAG.	DELTA(DA)
57.0708	MS2	9.769		
59.0612	MS2	1.915		
60.0816	MS2	1.632		
67.055	MS2	2.225		
69.0706	MS2	3.436	C5H9	0.0007
71.0737	MS2	3.055		
71.0862	MS2	9.569	C5H11	0.0007
81.0705	MS2	8.283	C6H9	0.0006
82.9454	MS2	1.124		
83.0862	MS2	4.69	C6H11	0.0007
85.1018	MS2	6.518	C6H13	0.0006
86.097	MS2	46.08	C5H12N	0.0006
87.1005	MS2	2.038	C5H12N [isotope]	1.004
88.0763	MS2	1.032		
95.086	MS2	10.507	C7H11	0.0005
96.0893	MS2	1.061	C7H11 [isotope]	1.0038
97.1016	MS2	3.296	C7H13	0.0004
109.1017	MS2	4.997	C8H13	0.0005
111.1172	MS2	1.65	C8H15	0.0004
118.0864	MS2	1.159		
119.0858	MS2	1.021		
123.1171	MS2	2.783	C9H15	0.0003

124.9999	MS2	2.469		
125.1329	MS2	1.01	C9H17	0.0005
137.1325	MS2	1.613	C10H17	0
137.6145	MS2	1.199		
140.9593	MS2	1.576		
146.9818	MS2	100	C2H5O4NaP	0.0001
147.9852	MS2	1.979	C2H5O4NaP [isotope]	1.0035
163.0154	MS2	1.391		
164.9925	MS2	1.065		
171.676	MS2	1.132		
184.0735	MS2	59.625		
185.077	MS2	2.051		
188.0084	MS2	3.61		
209.6056	MS2	1.078		
211.2061	MS2	2.824		
234.0656	MS2	1.239		
239.2367	MS2	1.456		
242.2465	MS2	1.057		
264.2678	MS2	1.094		
267.0589	MS2	1.248		
290.269	MS2	5.345		
291.2727	MS2	1.011		
311.0849	MS2	1.088		
336.1016	MS2	1.038		
391.224	MS2	1.129	M+H-N(CH3)3-FA(16:0)	-0.0004
413.2061	MS2	2.212	M+Na-N(CH3)3-FA(16:0)	-0.0002
441.2396	MS2	1.541	M+Na-N(CH3)3-FA(14:0)	0.0019
454.1466	MS2	1.096		
523.4716	MS2	16.214	NL[PC,+Na]+H	-0.0005
524.4736	MS2	3.935	NL[PC,+Na]+H [isotope]	1.0016
545.3803	MS2	1.494		
545.4532	MS2	24.733	NL[PC]	-0.0008
546.4562	MS2	6.639	NL[PC] [isotope]	1.0022
669.4458	MS2	22.07	M+Na-N(CH3)3	-0.0008
670.4505	MS2	12.112	M+Na-N(CH3)3 [isotope]	1.004
686.4384	MS2	16.396		
687.4409	MS2	5.82		
727.463	MS2	2.109		
728.5191	MS2	25.38	M+Na	-0.001
729.5224	MS2	12.242		

Table S11. Fragmentation pattern for lipids with rare occurrence in biological samples – PE 38:3 +Na+ in Hp analysis: M-score: 20.3, t-score: 0.6, Occupancy: 39.2

OBSMZ	TYPE	IT.(%)	FRAG.	DELTA(DA)
55.0551	MS2	4.209		
57.0344	MS2	1.002		
57.0707	MS2	16.545		
60.0816	MS2	1.139		
67.055	MS2	23.797		
69.0342	MS2	1.399		
69.0706	MS2	9.947		
71.0862	MS2	21.139	C5H11	0.0007
72.0895	MS2	1.046	C5H11 [isotope]	1.004
79.0548	MS2	15.341	C6H7	0.0006
81.0341	MS2	1.036		
81.0705	MS2	25.447	C6H9	0.0006

83.0497	MS2	2.101		
83.0861	MS2	10.927	C6H11	0.0006
85.1017	MS2	13.034	C6H13	0.0005
86.097	MS2	15.839		
86.1052	MS2	1.074	C6H13 [isotope]	1.004
87.1003	MS2	1.844		
91.0547	MS2	19.758		
92.058	MS2	1.52		
93.0704	MS2	17.181	C7H9	0.0005
94.0737	MS2	1.539	C7H9 [isotope]	1.0038
95.0496	MS2	1.367		
95.086	MS2	29.152	C7H11	0.0005
96.0893	MS2	1.831	C7H11 [isotope]	1.0037
97.0652	MS2	2.799		
97.1017	MS2	9.185	C7H13	0.0005
98.9847	MS2	1.029		
99.081	MS2	1.434		
99.1174	MS2	1.009		
104.1073	MS2	1.916		
105.0703	MS2	13.584		
106.0735	MS2	1.116		
107.0859	MS2	11.441	C8H11	0.0004
108.0893	MS2	1.179	C8H11 [isotope]	1.0038
109.0652	MS2	1.056		
109.1014	MS2	12.075	C8H13	0.0003
110.1049	MS2	1.499	C8H13 [isotope]	1.0037
111.1171	MS2	3.005	C8H15	0.0003
117.0701	MS2	8.548		
119.0858	MS2	14.356		
120.9663	MS2	15.636	H3O4P1Na1	0.0002
121.1014	MS2	7.596	C9H13	0.0002
123.1171	MS2	5.609	C9H15	0.0003
124.9377	MS2	33.579		
125	MS2	3.23		
129.0699	MS2	3.014		
131.0857	MS2	12.116		
132.0892	MS2	1.048		
133.1013	MS2	7.869		
135.117	MS2	4.418	C10H15	0.0001
136.9401	MS2	1.304		
137.1326	MS2	1.646	C10H17	0.0002
142.9481	MS2	8.606		
143.0855	MS2	4.194		
145.1012	MS2	5.803		
145.9975	MS2	1.222		
146.9819	MS2	3.754		
147.117	MS2	4.149	C11H15	0.0002
149.1327	MS2	1.586	C11H17	0.0002
151.1477	MS2	1.1	C11H19	-0.0004
155.0104	MS2	1.01		
155.0858	MS2	1.15		
157.1014	MS2	2.673		
159.1168	MS2	3.974		
161.1326	MS2	2.363	C12H17	0.0001
161.9718	MS2	1.372		
162.9558	MS2	6.434		
164.0084	MS2	15.238	(P-Etr)+Na	0
165.1637	MS2	1.228		
167.9799	MS2	52.2		
169.1013	MS2	1.092		

171.1167	MS2	3.448		
173.1325	MS2	1.993		
176.9924	MS2	1.239		
179.9822	MS2	10.894		
181.1016	MS2	1.069		
181.9804	MS2	1.537		
182.9795	MS2	4.621		
183.1169	MS2	1.732		
184.0735	MS2	86.786		
185.0769	MS2	7.792		
185.1324	MS2	1.332		
185.9904	MS2	16.513		
186.0778	MS2	2.228		
187.1484	MS2	1.125		
199.149	MS2	1.245		
201.1638	MS2	2.079		
209.1322	MS2	1.051		
211.1479	MS2	1.109		
226.0216	MS2	11.062		
262.9441	MS2	1.646		
264.2683	MS2	1.104		
267.268	MS2	2.466		
310.3106	MS2	2.801		
322.0177	MS2	4.096		
340.0281	MS2	6.211		
341.3052	MS2	7.243	MG(18:0)-OH	0.0002
342.308	MS2	3.564	MG(18:0)-OH [isotope]	1.003
354.2792	MS2	4.104		
365.3045	MS2	1.524		
373.2115	MS2	4.395		
385.2737	MS2	2.758		
387.2867	MS2	1.046		
389.3048	MS2	3.438		
404.2921	MS2	1.309		
421.2092	MS2	2.497		
447.3485	MS2	1.017		
650.5202	MS2	10.398		
651.534	MS2	100	NL[PE]	0.0017
652.5377	MS2	45.81	NL[PE] [isotope]	1.0054
653.5487	MS2	1.644		
732.4968	MS2	2.6		
733.5022	MS2	3.54		
748.4798	MS2	2.569		
749.4412	MS2	4.981		
751.4636	MS2	1.846		
791.5202	MS2	6.84		
792.49	MS2	57.233		
793.4983	MS2	31.23		
794.5058	MS2	13.149		

Table S12. Fragmentation pattern for lipids with rare occurrence in biological samples – PE O–34:1+Na+, which can also be PE P-36:3+ Na+; M-score: 21; T-score 0.4, Occurance: 34

OBSMZ	TYPE	IT.(%)	FRAG.	DELTA(DA)
55.0552	MS2	1.795		
57.0708	MS2	9.161		
59.0612	MS2	1.177		
67.055	MS2	13.978		
68.0584	MS2	1.416		

69.0707	MS2	7.931		
71.0862	MS2	9.076		
79.0549	MS2	9.063	C6H7	0.0007
80.9484	MS2	1.644		
81.0705	MS2	17.433	C6H9	0.0006
82.0738	MS2	1.689	C6H9 [isotope]	1.004
83.0862	MS2	6.646	C6H11	0.0007
84.0894	MS2	1.013	C6H11 [isotope]	1.0039
85.1018	MS2	8.91	C6H13	0.0006
86.097	MS2	10.219		
87.1004	MS2	1.007		
91.0548	MS2	11.589		
92.0582	MS2	1.148		
93.0704	MS2	12.404	C7H9	0.0005
95.0861	MS2	19.213	C7H11	0.0005
96.0894	MS2	1.5	C7H11 [isotope]	1.0039
97.0652	MS2	2.524		
97.1017	MS2	6.935	C7H13	0.0005
105.0703	MS2	6.399		
107.0859	MS2	6.98	C8H11	0.0004
109.1015	MS2	7.868	C8H13	0.0004
111.1172	MS2	2.521	C8H15	0.0004
117.0702	MS2	4.22		
119.0858	MS2	7.527		
120.0893	MS2	1.226		
120.9664	MS2	42.006	H3O4P1Na1	0.0003
121.1015	MS2	7.196	C9H13	0.0003
123.117	MS2	2.626	C9H15	0.0002
124.9378	MS2	48.123		
129.0701	MS2	2.721		
131.0858	MS2	5.515		
133.1014	MS2	4.375		
135.1169	MS2	3.317	C10H15	0.0001
136.9403	MS2	1.861		
137.1327	MS2	1.668	C10H17	0.0002
142.9482	MS2	12.961		
143.0857	MS2	1.343		
145.1012	MS2	2.792		
145.998	MS2	3.44		
146.9819	MS2	3.66		
147.1169	MS2	1.575	C11H15	0.0001
149.1326	MS2	1.508	C11H17	0.0001
157.1014	MS2	1.913		
159.1168	MS2	1.838		
161.1323	MS2	2.094	C12H17	-0.0001
161.9719	MS2	2.992		
162.9559	MS2	7.735		
164.0084	MS2	41.111	(P-Etr)+Na	0.0001
165.0118	MS2	2.94	(P-Etr)+Na [isotope]	1.0035
167.9798	MS2	84.274		
168.9832	MS2	1.383		
171.1167	MS2	1.372		
176.9924	MS2	1.204		
179.9824	MS2	15.546		
180.9859	MS2	1.254		
181.9805	MS2	2.936		
182.9796	MS2	6.615		
184.0735	MS2	35.941		
185.0771	MS2	2.197		
185.9904	MS2	26.035		

203.1797	MS2	2.302		
205.0351	MS2	1.829		
205.1948	MS2	1.141		
226.0217	MS2	14.827		
262.9438	MS2	1.018		
322.0173	MS2	1.303		
341.3053	MS2	7.12	MG(18:0)-OH	0.0003
342.3085	MS2	1.661	MG(18:0)-OH [isotope]	1.0034
354.2805	MS2	1.034		
365.3058	MS2	2.654		
373.2112	MS2	6.118		
374.2156	MS2	1.095		
421.2091	MS2	4.942		
443.2533	MS2	1.194		
608.5235	MS2	2.935		
609.5262	MS2	1.238		
628.5388	MS2	2.584		
629.5495	MS2	2.161	NL[PE,+Na]+H	-0.0008
649.5161	MS2	2.109		
650.5198	MS2	21.707		
651.5334	MS2	62.945	NL[PE]	0.0011
652.5374	MS2	30.389	NL[PE] [isotope]	1.0051
653.55	MS2	1.365		
668.5319	MS2	1.164		
732.4963	MS2	4.25		
733.5092	MS2	2.071		
748.4858	MS2	7.697		
749.4419	MS2	8.466		
750.4457	MS2	4.373		
751.4611	MS2	1.998		
791.5215	MS2	14.392		
792.4921	MS2	100		
793.4972	MS2	54.988		
794.5059	MS2	19.395		

Table S13. Fragmentation pattern for lipids with rare occurrence in biological samples – HexCer 43:1 +H+ in Hp analysis; M-score: 44.7; T-score: 0; Occupancy: 74.5

OBSMZ	TYPE	IT.(%)	FRAG.	DELTA(DA)
60.0452	MS2	2.355		
66.2176	MS2	1.181		
67.0114	MS2	1.135		
67.055	MS2	2.104		
69.0707	MS2	3.211		
72.8216	MS2	1.494		
79.0547	MS2	1.454		
81.0704	MS2	2.486		
82.0657	MS2	1.422		
85.029	MS2	1.527		
85.1016	MS2	1.415		
95.0861	MS2	4.562		
97.1016	MS2	2.563		
109.1017	MS2	3.16		
121.1016	MS2	1.871		
123.117	MS2	1.451		
135.117	MS2	1.887		
184.0736	MS2	1.958		
233.2386	MS2	1.386		

245.0189	MS2	1.313		
252.2687	MS2	16.71	So(d18:1)-H2O-CH2O	0.0002
253.2717	MS2	3.59	So(d18:1)-H2O-CH2O [isotope]	1.0031
256.2624	MS2	1.937		
263.6719	MS2	1.664		
264.2687	MS2	100	So(d18:1)-2H2O	0.0002
265.2721	MS2	20.031	So(d18:1)-2H2O [isotope]	1.0035
266.2736	MS2	1.812		
282.279	MS2	9.113	So(d18:1)-H2O	-0.0002
391.0856	MS2	1.329		
398.3968	MS2	3.303		
430.1158	MS2	1.658		
451.6485	MS2	1.394		
632.6345	MS2	2.385		
633.6378	MS2	3.203		
644.6326	MS2	17.512	NL[G1,H2O]	-0.0014
645.6365	MS2	7.403	NL[G1,H2O] [isotope]	1.0025
646.6473	MS2	2.386		
662.6442	MS2	9.377	NL[G1]	-0.0004
663.6477	MS2	7.054	NL[G1] [isotope]	1.0031
754.0703	MS2	1.613		
786.5255	MS2	1.766		
824.6978	MS2	2.539	NL[H2O]	0.0004
843.7356	MS2	1.71		

Table S14. Fragmentation pattern for lipids with rare occurrence in biological samples – HexCer 43:2;O2 +H+ in Hp analysis; M-score: 563.3; T-score: 2.3; Occupancy: 88.9

OBSMZ	TYPE	IT.(%)	FRAG.	DELTA(DA)
67.0551	MS2	1.879		
81.0705	MS2	2.243		
82.0657	MS2	2.726		
83.0861	MS2	1.117		
95.0861	MS2	2.805		
97.1016	MS2	1.365		
109.1016	MS2	1.538		
252.2686	MS2	10.706	So(d18:1)-H2O-CH2O	0.0001
253.2723	MS2	1.129	So(d18:1)-H2O-CH2O [isotope]	1.0038
262.253	MS2	2.019		
263.2565	MS2	1.086		
264.2687	MS2	100	So(d18:1)-2H2O	0.0002
265.2722	MS2	17.325	So(d18:1)-2H2O [isotope]	1.0036
282.2792	MS2	10.501	So(d18:1)-H2O	0.0001
283.2834	MS2	1.335	So(d18:1)-H2O [isotope]	1.0043
290.002	MS2	1.187		
343.0059	MS2	1.022		
551.5023	MS2	1.081		
626.624	MS2	5.32	NL[G1,H2O]	0.0006
627.6252	MS2	2.259	NL[G1,H2O] [isotope]	1.0017
628.6393	MS2	1.231		
644.6341	MS2	9.231	NL[G1]	0.0001
645.6369	MS2	3.774	NL[G1] [isotope]	1.0029
806.6857	MS2	5.071	NL[H2O]	-0.0011
807.6904	MS2	3.664	NL[H2O] [isotope]	1.0035
